# Vascular and Immune Cell Interactions in Atherosclerosis: Mechanisms, Plaque Destabilization, and Therapeutic Opportunities

**DOI:** 10.3390/cells15211927

**Published:** 2026-10-23

**Authors:** Yingli Zhou, Keying Xiao, Yuhua Chen, Yixuan He, Yan Liu, Wenyu Zhang, Yuyi Zhang, Zhaoyang Dong, Zhihua Guo, Jinxia Li, Jiaming Wei

**Affiliations:** 1 First Clinical College of Chinese Medicine, Hunan University of Chinese Medicine, Changsha 410208, China; zyl1998@stu.hnucm.edu.cn (Y.Z.); 004294@hnucm.edu.cn (Z.G.); 2 Hunan Key Laboratory of Colleges and Universities of Intelligent TCM Diagnosis and Preventive Treatment of Chronic Diseases, Hunan University of Chinese Medicine, Changsha 410208, China; 3 School of Chinese Medicine, Hunan University of Chinese Medicine, Changsha 410208, China

**Keywords:** atherosclerosis, cellular interactions, endothelial cells, vascular smooth muscle cells, macrophages, lymphocytes

## Abstract

**Highlights:**

**What are the main findings?**

• Atherosclerosis arises from dynamic, bidirectional interactions among four core cell types—endothelial cells, vascular smooth muscle cells, macrophages, and lymphocytes—rather than from dysfunction of any single cell type.
• Intercellular communication via paracrine signaling, exosome-mediated transfer, oxidative stress crosstalk, and autophagy regulation drives plaque microenvironment remodeling, lipid core expansion, fibrous cap degradation, and plaque destabilization.

**What are the implications of the main findings?**

• Targeting key cell–cell interaction nodes (e.g., the CX3CL1/CX3CR1 axis, CD40L–CD40 co-stimulation, and dual-targeting nanoplatforms) represents a promising strategy to stabilize vulnerable plaques.
• Deciphering cell–cell communication networks provides a theoretical basis for early screening, accurate diagnosis, and the development of novel targeted preventive and therapeutic strategies for atherosclerosis.

**Abstract:**

Atherosclerosis (AS) is a progressive vascular disease characterized by lipid accumulation within the arterial intima, chronic inflammation, and plaque formation. It represents the underlying pathological basis of major cardiovascular and cerebrovascular events, including coronary artery disease, acute myocardial infarction, and ischemic stroke. Accumulating evidence suggests that the initiation and progression of AS are not driven by dysfunction of a single cell type but rather result from complex and dynamic interactions among multiple vascular resident and immune cells, including endothelial cells (ECs), vascular smooth muscle cells (VSMCs), macrophages, and lymphocytes. This review systematically summarizes the functional and phenotypic characteristics of these four major cell types in atherosclerotic lesions, with particular emphasis on their bidirectional regulatory networks. We provide an in-depth analysis of key intercellular communication mechanisms, including paracrine signaling, exosome-mediated transfer, oxidative stress crosstalk, and autophagy regulation, all of which play central roles in plaque microenvironment remodeling, lipid core expansion, fibrous cap degradation, and plaque destabilization. Furthermore, we highlight recent advances in translational strategies targeting cell–cell interactions, focusing on the mechanisms of action and clinical potential of representative agents.

## 1. Introduction

Atherosclerosis (AS) is a chronic progressive vascular disease characterized by the gradual deposition of plaques in the arterial intima. As the core pathological basis of cardiovascular and cerebrovascular diseases, it is a leading cause of death worldwide and places a severe burden on public health systems. According to World Health Organization data, approximately 18 million people die annually from AS-related diseases, which can lead to serious complications such as coronary heart disease, acute myocardial infarction, ischemic stroke, and peripheral artery disease [1]. The management of AS has therefore become a major global health priority. The pathomorphological hallmark of the disease is the atherosclerotic plaque, which accumulates in large and medium-sized arteries through the deposition of lipids, cholesterol, calcium, fibrotic tissue, cells, and cellular debris [2]. Human blood vessels are structurally and functionally complex organs; arteries in particular comprise multiple cell populations, including endothelial cells (ECs), vascular smooth muscle cells (VSMCs), macrophages, and lymphocytes. Among these, VSMCs possess a unique capacity for phenotypic switching between contractile and synthetic states and play a central role in structural remodeling of the arterial wall. Excessive deposition of atherogenic lipids and lipoproteins in the arterial intima leads to structural disruption and dysfunction of ECs [3], marking the initiation of AS. Subsequently, the entire pathological process—from fatty streak formation to plaque progression and vulnerable plaque rupture—unfolds through a series of cellular interactions, including transcellular communication between ECs and VSMCs, phenotypic regulation of VSMCs by macrophages, inflammatory signal transfer among immune cells, and crosstalk between various cells and the plaque microenvironment [4]. Given the severity and urgency of AS prevention and control, effective intervention in this process has become a critical research focus. In this context, cell–cell interactions are key determinants of unstable plaque formation. This review explores the relevant pathogenic mechanisms, aiming to provide a theoretical basis for early screening and accurate diagnosis, and to inform the development of targeted preventive and therapeutic strategies, ultimately contributing to the reduction in morbidity and mortality associated with this disease.

## 2. Core Cell Types and Their Functions in AS

The atherosclerotic plaque microenvironment harbors a heterogeneous repertoire of vascular resident and immune cells, whose dynamic phenotypic plasticity and functional reprogramming underpin the entire pathological cascade of atherogenesis, lesion progression, and eventual plaque destabilization. Among these, ECs, VSMCs, macrophages, and lymphocytes emerge as the four core, functionally intertwined cell populations that collectively orchestrate the pathological remodeling of the arterial wall. Endothelial dysfunction constitutes the seminal initiating event of atherogenesis; VSMCs serve as the principal mediators of vascular structural remodeling; macrophages govern innate inflammatory responses and intraplaque lipid homeostasis; and lymphocytes orchestrate antigen-specific adaptive immune regulation (Figure 1).

### 2.1. ECs

ECs form a continuous, densely arranged monolayer of flattened ECs lining the vascular wall, with intact cell membranes and intercellular junctions. Their primary physiological functions include forming the vascular barrier, regulating permeability, sensing shear stress from blood flow, synthesizing and releasing vasoactive substances such as nitric oxide (NO), and participating in the regulation of inflammatory adhesion, coagulation balance, and vascular remodeling. They are critical for maintaining vascular homeostasis. In AS, endothelial dysfunction contributes to the pathological process through multiple mechanisms. For example, under conditions of barrier impairment, abnormal blood flow shear stress further disrupts endothelial barrier integrity, increasing permeability and facilitating the entry of lipoproteins and other substances into the intima. With regard to inflammatory immune cell recruitment, oxidized lipoproteins stimulate ECs to upregulate adhesion molecules and chemokines, promoting local recruitment and infiltration of monocytes and other inflammatory cells, thereby driving lipid deposition and foam cell formation. Additionally, endothelial phenotypic transformation—namely endothelial-to-mesenchymal transition (EndMT)—leads to loss of endothelial characteristics, which not only exacerbates barrier dysfunction but also promotes structural remodeling of the vascular wall.

In healthy vessels, ECs exhibit low metabolic activity and remain in a quiescent state, thereby maintaining vascular integrity, regulating permeability, and preventing inappropriate coagulation and inflammation. With aging and the gradual accumulation of stimuli such as inflammatory cytokines, growth factors, and mechanical stress, ECs undergo activation, leading to a series of functional alterations [5]. On the one hand, disruption of endothelial integrity and tight junctions results in increased vascular permeability and impaired endothelial homeostasis, facilitating the transendothelial transport of plasma components, low-density lipoprotein (LDL), and triglyceride-rich lipoproteins [6]. Notably, shear stress—the frictional force generated by blood viscosity and tangential flow on the endothelial surface—is the predominant hemodynamic force acting on ECs [7]. Shear stress is a key regulator of endothelial function, influencing cell fate determination, permeability, and extracellular matrix production. Laminar flow generally exerts atheroprotective effects by enhancing junctional protein expression and signaling, thereby strengthening tight junctions and adherens junction integrity [8]. In contrast, disturbed or oscillatory flow induces pro-atherogenic endothelial responses, including cellular senescence, apoptosis, upregulation of adhesion molecules, and increased permeability. On the other hand, endothelial activation promotes inflammatory and immune cell recruitment by upregulating adhesion molecules and chemokines, thereby mediating the adhesion and infiltration of myeloid cells. These cells accumulate within developing atherosclerotic lesions and engulf modified lipids, eventually differentiating into foam cells during early fatty streak formation [9]. In addition, oxidized lipoproteins and other inflammatory mediators further amplify endothelial activation, resulting in increased expression of P-selectin, E-selectin, VCAM-1, and ICAM-1, which promotes adhesion of CCR2-expressing monocytes and other leukocytes. Disturbed flow may also induce superficial endothelial erosion via TLR2-dependent endothelial apoptosis and IL-8 secretion, leading to neutrophil recruitment and activation, neutrophil extracellular trap (NET) formation, endothelial injury, and potentially thrombosis [6]. Furthermore, as the primary barrier of the vascular wall, ECs play a critical role in preventing the entry of pathogenic substances into the subendothelial space. A key phenotypic transition involved in this process is endothelial-to-mesenchymal transition (EndMT) [10], during which ECs lose characteristic markers such as platelet endothelial cell adhesion molecule-1 (PECAM-1/CD31) and acquire mesenchymal-like features and migratory capacity. EndMT contributes to endothelial dysfunction, increases vascular permeability, and facilitates the infiltration of lipids, metabolic waste, cytokines, and other pathological factors into the subendothelial region, thereby promoting the development of cardiovascular lesions [11,12,13].

### 2.2. VSMCs

VSMCs are the predominant cell type of the arterial media, exhibiting a spindle-shaped morphology and remarkable phenotypic plasticity, and they play a central role in preserving vascular structural integrity and functional homeostasis. Under physiological conditions, VSMCs mainly adopt a contractile phenotype, participating in blood pressure regulation by modulating vascular tone while secreting small amounts of extracellular matrix (ECM) to maintain the structural integrity and elasticity of the arterial wall [14,15]. In AS, functional alterations in VSMCs affect plaque formation and stability primarily through two mechanisms: phenotypic switching and inflammatory responses. Phenotypic switching can be broadly categorized into several directions, including fibrosis, calcification, and lipid accumulation. In the fibrotic direction, VSMCs dedifferentiate into a synthetic phenotype and participate in fibrous cap formation and remodeling; when the repair capacity of the cap is impaired, plaques become prone to rupture. In the calcification direction, VSMCs transdifferentiate into osteoblast-like cells, promoting calcium salt deposition, which leads to vascular wall stiffening and dysfunction. In the lipid accumulation direction, VSMCs transform into foam cells and represent a major source of foam cells in advanced plaques; their apoptosis further promotes necrotic core formation, increasing plaque vulnerability [16]. In terms of inflammatory response, VSMCs act both as recipients of inflammatory stimuli and as active participants in the inflammatory process; upon stimulation by pro-inflammatory factors, they can switch to a pro-inflammatory phenotype, secreting chemokines that recruit immune cells, thereby establishing a vicious inflammatory cycle that exacerbates plaque instability.

In the resting state, VSMCs are embedded within the matrix of the tunica media and primarily function to regulate intravascular pressure and blood flow distribution. Upon stimulation by various endogenous and exogenous factors, a series of functional disturbances occurs. On the one hand, with respect to phenotypic switching, contractile VSMCs dedifferentiate, lose expression of typical smooth muscle cell markers, and enter a highly plastic state. Under the influence of transforming growth factor-β (TGF-β) signaling, they convert to a fibrotic phenotype (SMCs-F) [17]. The ECM secreted by VSMCs forms the fibrous cap of the plaque; cap thickness and collagen content are critical determinants of plaque stability. When the VSMC function is intact and collagen synthesis is sufficient, the fibrous cap remains dense and resilient, conferring high plaque stability. Conversely, when oxidized low-density lipoproteins (ox-LDL) and inflammatory cytokines persistently damage VSMCs, leading to increased apoptosis or decreased collagen synthesis, the cap becomes thin and fragile, predisposing to plaque rupture. In the calcification process, calcification can occur in the intima, media, and valves. Intimal calcification is a core manifestation of atherosclerotic lesions, whereas medial calcification is primarily driven by VSMCs. Within the pathological microenvironment, VSMCs may lose contractile markers and undergo phenotypic transition toward chondrocyte-, osteoblast-, and osteocyte-like phenotypes [18], secreting calcification-related proteins such as osteopontin and osteocalcin, which promote calcium salt deposition, leading to plaque calcification and arterial stiffness, and further aggravating vascular dysfunction. Additionally, VSMCs may exhibit lipid overload or transform into a foam cell state, a phenotype mainly driven by cholesterol and ox-LDL [17]. ox-LDL accumulated in the intima chemoattracts monocytes, which differentiate into macrophages. Notably, both VSMCs and macrophages give rise to lipid-laden foam cells, with VSMC-derived foam cells contributing a substantial proportion of all foam cells in advanced human coronary plaques [19]. These transformed foam cells undergo apoptosis, further promoting necrotic core formation and inflammation. Lipid accumulation and persistent inflammation ultimately produce vulnerable plaques characterized by a large necrotic core and a thin fibrous cap, increasing the risk of plaque rupture, thrombosis, and sudden death [20]. On the other hand, although VSMCs have traditionally been considered passive victims of inflammatory mechanisms, recent studies have confirmed that they are active inflammatory participants. VSMC apoptosis frequently occurs in regions with high inflammatory activity, suggesting that inflammatory cells or pro-inflammatory cytokines may sensitize VSMCs to apoptosis [21]. Indeed, unlike many cell types, VSMCs sequester the death receptors Fas/TNFR1 in the Golgi apparatus, rendering them relatively resistant to Fas-induced apoptosis [22]. Upon specific stimulation, such as pro-inflammatory cytokines (IL-1β, TNF-α, and IFN-γ), ox-LDL, NO, and free radicals, Fas can be redistributed to the cell surface, initiating VSMC death [23]. Furthermore, after inflammatory stimulation, VSMCs activate the NF-κB/MAPK pathway, shifting to a pro-inflammatory phenotype characterized by downregulation of smooth muscle myosin heavy chain (SM-MHC), upregulation of vimentin and ICAM-1, and subsequent secretion of monocyte chemoattractant protein-1 (MCP-1), IL-6, and IL-1β. These factors recruit macrophages and T cells, which in turn release additional inflammatory cytokines, further activating VSMC inflammatory responses and perpetuating a vicious cycle [24].

### 2.3. Macrophages

Macrophages (Mφ) originate from bone marrow hematopoietic stem cells and differentiate from monocytes [25]; they are widely distributed and play important roles in maintaining tissue homeostasis, clearing apoptotic cells and debris, and regulating inflammation. In atherosclerotic plaques, macrophages are classified into M1 (pro-inflammatory) and M2 (anti-inflammatory) phenotypes [26]. M1 macrophages are induced by Th1-derived IFN-γ or ox-LDL and secrete large amounts of pro-inflammatory cytokines, driving inflammatory amplification. M2 macrophages respond to Th2-type cytokines, secrete IL-10 and TGF-β, and exert protective effects in inflammation resolution and tissue repair. Additional subtypes, such as CXCL4-induced M4 macrophages, also exist within plaques, highlighting the high heterogeneity of macrophages [27]. Under metabolic derangement, M2 macrophages can shift toward an M1 phenotype, with protective functions being supplanted by pro-inflammatory activities; this imbalance in metabolic reprogramming drives pro-atherogenic polarization. Thus, macrophages possess dual pro-inflammatory and anti-inflammatory functions: the pro-inflammatory phenotype takes up ox-LDL to form foam cells, secretes pro-inflammatory cytokines and matrix-degrading enzymes, thereby promoting plaque destabilization; the anti-inflammatory phenotype produces IL-10 and TGF-β, promotes collagen deposition and fibrous cap thickening, and maintains plaque stability through efferocytosis [28].

M1 macrophages drive the initiation, progression, and destabilization of atherosclerotic plaques through lipid uptake, inflammatory factor secretion, and ECM degradation. Regarding lipid uptake and foam cell formation, M1 macrophages internalize ox-LDL via scavenger receptors (SRs) [29]; scavenger receptor A (SR-A) and cluster of differentiation 36 (CD36) are two key receptors mediating this process [30]. CD36-dependent signaling activates Src kinase and c-Jun N-terminal kinase (JNK), promoting ox-LDL uptake and foam cell formation [31]. Studies have shown that M1 macrophages are more prone to lipid uptake and foam cell transformation than M2 macrophages, and this process is accompanied by sustained activation of the NF-κB signaling pathway [32]. With respect to inflammatory cytokine secretion, activated M1 macrophages promote TNF-α synthesis and release via the NF-κB pathway, and depend on the NLRP3 inflammasome pathway to cleave and secrete mature IL-1β [33,34], thus forming a local positive-feedback amplification loop. M1 macrophages also secrete various chemokines, such as CCL4 [35], further recruiting circulating monocytes to the plaque area and exacerbating lesion progression [36]. In terms of ECM degradation, M1 macrophages overexpress multiple matrix metalloproteinases (MMPs), including MMP-2, MMP-9, MMP-12, MMP-13, and MMP-14; among these, collagenases degrade collagen fibers in the fibrous cap, a key structural component maintaining plaque mechanical strength [37]. MMP-9 and MMP-14 directly weaken the mechanical strength of the fibrous cap by degrading basement membrane and collagen fibers, rendering the plaque susceptible to a vulnerable phenotype and rupture [38,39]. In M1 macrophages, proprotein convertase subtilisin/kexin type 9 (PCSK9) further promotes the expression of pro-inflammatory factors such as TNF-α and IL-1β, exacerbating inflammation [40]. Additionally, M1 macrophages participate in necrotic core formation: as foam cells accumulate and eventually undergo apoptosis and necrosis, cellular debris and lipids together form the necrotic core, further reducing plaque stability and increasing the risk of thrombosis [41,42].

M2 macrophages exert protective effects by secreting anti-inflammatory factors, promoting fibrosis, and performing efferocytosis, thereby stabilizing plaques. Their enrichment during the resolution phase depends on the recruitment of Ly6Chi monocytes and STAT6-dependent polarization. With respect to anti-inflammatory cytokine secretion, M2 macrophages suppress local inflammatory responses and promote inflammation resolution by secreting IL-10 and TGF-β [43]. IL-10 reduces plaque area and necrotic core size, whereas TGF-β uniquely suppresses inflammation by reducing inducible nitric oxide synthase (iNOS) activity. The M2 marker arginase-1 (Arg-1) converts L-arginine to L-ornithine, a precursor for collagen synthesis and VSMC differentiation, and simultaneously limits the availability of the substrate L-arginine, thereby reducing iNOS-mediated cytotoxic NO overproduction, alleviating local nitrosative stress, and stabilizing the fibrous cap [44,45]. In terms of cell clearance, M2 macrophages efficiently clear apoptotic cells and debris through efferocytosis; this process triggers pro-resolving signals and releases TGF-β and IL-10, thereby reducing plaque area, limiting necrotic core expansion, and enhancing structural stability. Furthermore, enhancing STAT6-dependent M2 polarization of Ly6Chi inflammatory monocytes or targeting CCR2/CX3CR1-mediated monocyte recruitment pathways can promote M2 macrophage enrichment within plaques, thus facilitating inflammation resolution and plaque regression [46,47]. Studies have shown that following normalization of blood lipid levels, plaque regression is driven not by the phenotypic reversal of pre-existing macrophages within the plaque, but by the continuous recruitment of circulating Ly6Chi inflammatory monocytes. These monocytes enter regressing plaques via CCR2 and CX3CR1 and, under the influence of the local microenvironment, polarize into M2 macrophages through STAT6-dependent signaling [48]. Deletion of CCR2 or CX3CR1 leads to regression failure, whereas deletion of CCR5 has no effect, confirming that Ly6Clo patrolling monocytes are not essential. Acute inhibition of CCR2 prevents plaque regression and M2 enrichment, and STAT6 deficiency impedes M2 accumulation, thereby impairing regression [46].

### 2.4. Lymphocytes

Lymphocytes are the core cell population of adaptive immunity and are mainly divided into T lymphocytes (T cells) and B lymphocytes (B cells). They are characterized by high antigen specificity and immunological memory, maintaining immune homeostasis through cellular and humoral immunity while eliminating pathogens and abnormal cells. In atherosclerotic lesions, lymphocytes cooperate with innate immune cells to shape the immune microenvironment, imparting memory and antigen-specific features to AS immunopathology [49]. Single-cell RNA sequencing of vascular CD45^+^ leukocytes identifies T cells as a major leukocyte subset in the aorta; by contrast, within established plaques, macrophages and other myeloid cells predominate, while B cells reside mainly in the adventitia and artery tertiary lymphoid organs, indicating that the reported proportions depend on the sampling site and platform [50]. Overall, lymphocytes may influence the AS immune microenvironment through both the cytokine network of T-cell subsets and the antibody- and chemokine-mediated regulation by B-cell subsets. The functional imbalance between effector T cells and regulatory T cells (Tregs), as well as dynamic changes in the B1/B2 subset ratio, are likely to play important roles in the development and clinical outcomes of AS.

T-cell subsets exert differential regulatory effects in AS. CD4^+^ T cells in the local microenvironment can differentiate into functionally distinct effector subsets, including Th1 cells characterized by IFN-γ secretion [51], Th17 cells producing IL-17, and Tregs that express Foxp3 and maintain immune tolerance through IL-10 and TGF-β secretion [52,53]. Th1 cells represent the most abundant CD4^+^ T-cell subtype in plaques; T-cell clones derived from human aortic atherosclerotic plaques predominantly secrete IFN-γ, underscoring their central role in regulating macrophage and VSMC functions [54]. IFN-γ activates the STAT1 and NF-κB pathways, significantly upregulating VCAM-1 and ICAM-1 expression to promote monocyte recruitment, while simultaneously inhibiting collagen synthesis in VSMCs, thereby weakening fibrous cap strength [55]. IFN-γ activates macrophages and promote foam cell formation, accelerating lipid core expansion [56]. Th17 cells characteristically secrete IL-17, which plays a context-dependent role in AS: on the one hand, it promotes neutrophil recruitment and exacerbates inflammation; on the other hand, in TRIM21-deficient mice, IL-17 enhances collagen deposition and plaque stability [57,58]. Tregs inhibit effector T-cell activation through IL-10 and TGF-β; however, during AS progression, Tregs can undergo phenotypic conversion to Th1-like Tregs secreting IFN-γ, thereby losing their immunosuppressive function and aggravating arterial inflammation [47]. The imbalance between effector T cells and Tregs is a key factor in plaque destabilization; in patients with acute coronary syndromes (ACSs), CD4^+^CD28^−^ T cells are significantly increased while Tregs are decreased, a shift that correlates with fibrous cap thinning and an elevated risk of rupture [52,59].

B-cell subsets also display marked functional heterogeneity. B1 cells primarily exert atheroprotective effects by secreting natural IgM antibodies, whereas B2 cells may aggravate inflammation through antigen presentation and the secretion of pro-inflammatory cytokines [60]. B1 cells continuously produce natural IgM antibodies that specifically recognize oxidized epitopes on ox-LDL, thereby blocking ox-LDL uptake by ECs and macrophages, inhibiting foam cell formation, and promoting the clearance of apoptotic cells, thus reducing the intra-plaque inflammatory burden [61,62]. Clinical studies have shown that plasma levels of IgM antibodies against malondialdehyde-modified LDL (MDA-LDL) are significantly inversely associated with the incidence of coronary artery disease and cardiovascular events [63]. In contrast, B2 cells activate pathogenic T cells via antigen presentation, secrete pro-inflammatory cytokines, and produce ox-LDL-specific IgG antibodies; these antibodies form immune complexes that deposit on the vessel wall, thereby amplifying local inflammation through complement activation and Fcγ receptor engagement. Selective depletion of B2 cells attenuates AS, whereas total B-cell deficiency worsens lesions, further confirming the functionally distinct roles of these subsets. The CXCL13/CXCR5 axis mediates B-cell homing to the adventitia and drives the formation of artery tertiary lymphoid organs (ATLOs), thereby establishing organized adaptive immune centers within the adventitia, extending the local immune response from the intima to the adventitia, and significantly amplifying local adaptive immune reactions [64,65].

## 3. Cellular Interactions and Their Relationship to AS

### 3.1. ECs and VSMCs

The interaction between ECs and smooth muscle cells encompasses both direct cell–cell contact and indirect signal transduction, forming a complex communication network that is essential for maintaining vascular homeostasis. Under physiological conditions, this bidirectional dialogue ensures vasomotor balance and structural integrity; under pathological conditions such as AS, disruption of this communication network becomes a key driver of disease initiation and progression. With respect to modes of action, direct contact mainly involves Notch signaling (Jagged1/Dll4–Notch1/3) and gap junctions (Cx37/Cx40), which regulate VSMC differentiation and maturation [66,67]. Indirect signal transduction is primarily achieved through paracrine pathways, with the involvement of multiple cytokines and growth factors, including TGF-β, platelet-derived growth factor (PDGF), and MCP-1 family members [68,69,70], which together constitute the fundamental regulatory framework of endothelial–smooth muscle communication. This regulation is not unidirectional; VSMCs can also provide feedback regulation on EC function via paracrine pathways, forming a complete bidirectional communication network. Under physiological conditions, this network maintains vascular homeostasis; during AS, its imbalance can generate positive-feedback mechanisms that accelerate lesion progression.

Under pathological conditions, ECs exert dual regulatory effects on VSMCs through paracrine pathways, with pro-atherogenic effects playing a particularly critical role [71]. Classical mechanisms include the MCP-1/CCR2 axis mediating monocyte recruitment and VSMC migration, members of the PDGF family promoting VSMC proliferation and dedifferentiation, and TGF-β/SMAD pathway-mediated vascular remodeling under pathological stimulation [72,73]. Disturbed flow induces ER stress in ECs, which promotes TGF-β1 expression via the PERK-ATF4 pathway; TGF-β1 in turn activates VSMCs and induces phenotypic switching in a paracrine manner [74,75,76]. Under disturbed flow, polycystin-1 in ECs not only regulates CTGF expression through the AKT pathway but also modulates the switching of VSMCs to a synthetic phenotype [77], suggesting that the PKD1-AKT-CTGF axis may represent a novel mechanism of endothelial–smooth muscle paracrine communication [78]. Moreover, paracrine factors released by ECs in response to oscillatory shear stress—including CTGF and PDGF—induce VSMC conversion to a synthetic phenotype and promote proliferation, accompanied by a significant upregulation of ECM synthesis and downregulation of contractile markers such as α-smooth muscle actin (α-SMA) [79,80], indicating that hemodynamic factors play an important regulatory role in this communication. Furthermore, non-coding RNA-mediated regulation provides higher-order fine-tuning of this paracrine network. Circulating microRNAs play key roles in the pathology of coronary artery ectasia (CAE) by modulating angiogenesis and AS-related pathways. Studies have shown that plasma miR-126 and miR-145 are significantly upregulated in CAE patients, while miR-24-1-5p is downregulated, suggesting specific associations with abnormal coronary dilation [81]. Mechanistically, miR-126 and miR-145 are key regulators that maintain the VSMC contractile phenotype, capable of inhibiting target genes such as KLF5 and PI3K, thereby blocking VSMC conversion to the synthetic/dedifferentiated phenotype [82,83]. Additionally, other non-coding RNAs secreted by ECs, such as lncRNA SENCR, miR-155, and miR-146, also play important roles in modulating VSMC function [84]. Counterbalancing these pro-atherogenic effects, ECs also maintain the VSMC contractile phenotype and exert vascular protection through miRNA signaling, extracellular vesicle/exosome-mediated molecular transfer, and direct paracrine factor actions. For example, endothelial-derived extracellular vesicles (EVs) carrying miR-145 can be transferred to VSMCs, thereby blocking their conversion to the synthetic phenotype [85,86,87,88]. In vascular injury models, endothelial-specific miR-126 knockout does not affect normal vascular wall structure but significantly exacerbates neointima formation [89]. Moreover, treatment of VSMCs with conditioned medium from ECs overexpressing miR-212 significantly suppresses VSMC proliferation and migration and decreases the expression of the synthetic markers OPN and PCNA, indicating that miR-212 attenuates the transmission of EC-derived pro-proliferative signals to VSMCs [90]. Endothelial-derived exosomes overexpressing miR-145 alleviate PDGF-BB-induced VSMC proliferation and phenotypic switching [91]. Additionally, studies have shown that angiopoietin-1 (Ang-1), fibroblast growth factor-2 (FGF-2), and NO modulate molecular communication between ECs and VSMCs [92,93].

VSMCs also provide feedback regulation of EC function via paracrine pathways, representing another key aspect of bidirectional communication. Under physiological conditions, VSMC-derived exosomes can transfer miR-221 to ECs, where they modulate endothelial signalling, such as the PTEN/Akt pathway, and promote a pro-atherogenic endothelial phenotype [94]. In addition, VSMC-secreted VEGF and hepatocyte growth factor (HGF) significantly stimulate EC proliferation and re-endothelialization, which not only accelerates vascular repair after injury but also enhances barrier function and helps maintain vascular homeostasis [95]. Thus, under physiological conditions, VSMCs regulate EC migration, proliferation, and function through the secretion of soluble factors, exosomes, and microRNAs, thereby indirectly influencing vascular remodeling and elasticity and preserving overall vascular health. However, in the pathological microenvironment of AS, the feedback effects of VSMCs exhibit distinct characteristics: their secretion of abnormal cytokines, such as TGF-β and inflammatory mediators, can disrupt endothelial tight junctions, leading to markedly increased vascular permeability and enhanced lipid infiltration [96]. Concurrently, VSMC-secreted PDGF-BB induces the expression of endothelial adhesion molecules, promoting monocyte adhesion to the endothelium and subsequent transmigration into the vessel wall, thereby establishing a positive-feedback loop [97]. Beyond these effects, VSMCs under pathological conditions may further influence EC function through the secretion of inflammatory mediators, modulation of oxidative stress, and disruption of intercellular tight junctions, resulting in inflammatory activation, restricted migration, and barrier dysfunction in ECs, which collectively accelerate the progression of AS (Figure 2).

### 3.2. ECs and Macrophages

The bidirectional regulatory network between ECs and macrophages, mediated by multiple signaling pathways, constitutes a core inflammatory mechanism driving the initiation and progression of AS. Under pathological conditions, endothelial dysfunction triggers immune cell recruitment and activation, while macrophages further modulate EC function through inflammatory amplification and tissue remodeling, jointly promoting plaque formation and progression. ECs regulate macrophage recruitment and polarization via chemokines, cytokines, and exosomes, whereas macrophages, in turn, regulate EC function through inflammatory factors, reactive oxygen species (ROS), matrix metalloproteinases, and exosomes, forming a complete feedback loop. Under physiological conditions, macrophages help maintain EC homeostasis and promote vascular repair by secreting growth factors and anti-inflammatory mediators; however, in the atherosclerotic microenvironment, disruption of this network accelerates lesion progression through inflammatory amplification.

In early AS, ECs respond to abnormal shear stress, hyperlipidemia, and oxidative stress by becoming dysfunctional and initiating inflammatory responses [98]. Injured ECs first secrete chemokines such as MCP-1 and upregulate adhesion molecules (VCAM-1, ICAM-1), thereby promoting monocyte migration into the intima and their differentiation into macrophages [99]. In addition, EC-derived CCL2, CX3CL1, CCL5, and other chemokines selectively recruit distinct macrophage subsets in different vascular regions [100]. Following local recruitment, ECs further regulate macrophage polarization by secreting cytokines such as TGF-β and VEGF, thereby influencing their conversion toward pro-inflammatory (M1) or anti-inflammatory (M2) phenotypes [101]. Differentially polarized macrophages exert distinct effects on inflammatory responses and plaque stability regulation, thus shaping the course of AS. Within the sustained inflammatory environment, macrophages take up ox-LDL via SRs, gradually transform into foam cells, and contribute to the formation of the plaque lipid core [29]. This process is co-regulated by EC-derived factors and the local inflammatory microenvironment and represents a critical step in atherosclerotic progression. Beyond these classical mechanisms, ECs can also mediate transcellular communication by transferring exosomal miRNAs to macrophages. For example, endothelial-derived exosomal miR-92a induces a pro-inflammatory macrophage phenotype, whereas miR-126 inhibits macrophage infiltration and attenuates plaque formation [102,103]. Moreover, multiple miRNAs modulate macrophage function by targeting the NF-κB, STAT, and other signaling pathways [104].

Conversely, macrophages exert significant feedback regulation on ECs. First, through inflammatory amplification, macrophages secrete pro-inflammatory cytokines such as TNF-α and IL-1β, which upregulate endothelial adhesion molecule expression (ICAM-1, VCAM-1), enhance monocyte adhesion and transendothelial migration, and thereby create an inflammatory cascade amplification effect [105]. These cytokines further augment local inflammation via the NF-κB pathway and related mechanisms [106]. Second, macrophages secrete matrix metalloproteinases (MMPs) that degrade the ECM, compromising vascular structural stability and disrupting endothelial barrier integrity, which renders vessels more susceptible to inflammatory stimuli and facilitates lipid deposition and further inflammatory cell infiltration. Third, in the context of oxidative stress, macrophage-derived ROS reduce NO bioavailability, induce endothelial dysfunction, and further aggravate inflammation [107], particularly under hyperglycemic and oxidative conditions. Additionally, macrophage-secreted exosomes participate in EC regulation: exosomal miR-150 promotes EC migration, while miR-155 induces endothelial inflammatory responses and dysfunction [103]. Taken together, macrophages regulate EC function through multiple pathways, establishing a positive-feedback loop. Under physiological conditions, they help maintain endothelial homeostasis and support vascular repair through growth factors and anti-inflammatory mediators. However, in the pathological atherosclerotic microenvironment, the abundant release of inflammatory cytokines and ROS by macrophages induces endothelial dysfunction, increases endothelial permeability, and upregulates adhesion molecule expression, thereby exacerbating inflammatory cell recruitment and lipid deposition and further amplifying the pathological process (Figure 3).

### 3.3. ECs and Lymphocytes

The bidirectional regulatory network between ECs and lymphocytes, mediated by multiple signaling pathways, is an essential component of adaptive immune responses in AS. Unlike innate immune cells such as macrophages, lymphocyte-mediated immune responses are characterized by specificity and memory, playing important roles in regulating vascular inflammation and plaque stability. ECs modulate lymphocyte recruitment and functional status through chemokines, immunoregulatory factors, and exosomes, whereas lymphocytes regulate ECs via the release of inflammatory cytokines and immune cascades, thereby participating in both the initiation and progression of AS. Under physiological conditions, this interaction helps maintain vascular immune homeostasis; during AS, its dysregulation sustains chronic inflammation and drives lesion progression.

During atherogenesis, ECs undergo functional changes in response to inflammatory stimuli and hemodynamic alterations. ECs first secrete various chemokines (such as CCL2, CXCL9, CXCL10, and CXCL13) and upregulate adhesion molecules (ICAM-1, VCAM-1), thereby promoting lymphocyte recruitment and adhesion to the intima [108,109]. These distinct chemokines selectively recruit T and B cell subsets, contributing to the establishment of the local immune microenvironment. Following lymphocyte recruitment, ECs further influence their activation and differentiation through the secretion of immunoregulatory factors. For instance, TGF-β promotes the differentiation of Tregs and maintains their suppressive function, thus exerting local immunoprotective effects [110]; meanwhile, VEGF and other factors increase vascular permeability and modulate lymphocyte activation, facilitating their migration to inflammatory sites [111]. Through these mechanisms, ECs dynamically regulate the balance between immune activation and suppression. Additionally, EC-derived NO not only sustains vasodilation but also indirectly inhibits excessive lymphocyte activation by suppressing NF-κB signaling and inflammatory factor expression, thereby contributing to vascular immune homeostasis [112]. Beyond classical soluble mediators, ECs can mediate transcellular communication via exosomes, transferring miRNAs (such as miR-10a, miR-126, and miR-92a) to lymphocytes to regulate their migration, proliferation, and phenotypic conversion [113]. Different miRNAs target specific transcription factors and signaling pathways, further refining this regulatory process.

Conversely, lymphocytes exert significant feedback regulation on ECs. With regard to inflammatory amplification, activated T cells (e.g., Th1, Th17) and NK cells secrete cytokines such as IFN-γ, IL-17, and TNF-α, which upregulate endothelial VCAM-1 and ICAM-1 expression via NF-κB and other pathways, thereby enhancing immune cell adhesion and transendothelial migration and establishing an amplification effect [114,115]. Sustained lymphocyte activation can trigger immune cascades characterized by the continuous release of inflammatory mediators, which recruit and activate additional immune cells and generate a positive-feedback loop that persistently amplifies the local inflammatory environment [116,117]. In terms of vascular remodeling, lymphocyte-derived cytokines and matrix-regulatory proteins (e.g., MMPs) participate in structural alterations of the vessel wall, affecting endothelial barrier function and plaque stability [118]. Studies have shown that the degree of lymphocyte activation is closely associated with fibrous cap stability, with aberrant activation increasing the risk of plaque rupture [119]. Additionally, lymphocyte-derived exosomes contribute to the feedback regulation of ECs, miR-30d-5p promotes endothelial inflammation via NF-κB activation, while miR-21a-5p and other exosomal miRNAs modulate the inflammatory status and functional phenotype of ECs in a multidimensional manner [120,121] (Figure 4).

### 3.4. VSMCs and Macrophages

In the pathological process of AS, characterized by chronic inflammation and vascular wall remodeling, the bidirectional crosstalk between activated macrophages (Mφ) and VSMCs serves as a central node that reshapes the plaque microenvironment, modulates inflammatory levels, and determines plaque stability. These two cell types establish a complex regulatory network through paracrine signaling, exosome transfer, oxidative stress crosstalk, and autophagy interactions, collectively contributing to intimal hyperplasia, lipid deposition, and destruction of vascular architecture, thereby profoundly influencing the pathological progression of AS. Macrophages drive aberrant VSMC activation and intimal hyperplasia via these pathways, while VSMCs reciprocally regulate macrophage recruitment, polarization, and function through the same mechanisms, forming a bidirectional positive-feedback pathological loop.

Under pathological conditions, activated macrophages systematically regulate VSMCs through paracrine, exosome, oxidative stress, and autophagy pathways, jointly driving VSMC phenotypic switching, proliferation, migration, and functional derangement. Classical paracrine mechanisms include the release of PDGF and MCP-1 by activated macrophages, which directly activate corresponding signaling axes in VSMCs, promoting their transition from a contractile to a highly proliferative, migratory synthetic phenotype and mediating the migration of medial VSMCs into the intima, thereby driving intimal hyperplasia and plaque expansion [122]. When complement C1q A chain (C1QA) expression is downregulated in macrophages, M1 pro-inflammatory polarization is enhanced, accompanied by massive secretion of TNF-α, IL-1β, and IL-6, which further upregulate VSMC migration and proliferation through intracellular signaling cascades, exacerbating inflammatory infiltration and abnormal intimal hyperplasia [123]. Exosomes, a major subtype of extracellular vesicles (EVs), serve as important carriers for long-distance intercellular communication, significantly expanding the regulatory dimension. Under sustained lipid loading and inflammatory stress, activated macrophages secrete exosomes of 30–150 nm in diameter that carry pro-inflammatory microRNAs (miR-155, miR-34a), pro-inflammatory proteins, and oxidized lipids. These bioactive cargos enable remote signal transmission within the plaque microenvironment without direct cell contact; upon uptake by VSMCs, they induce phenotypic switching, enhance proliferation and migration, and accelerate local inflammatory amplification [124]. Concurrently, oxidative stress constitutes a critical link between macrophage activation and VSMC dysfunction. Pathologically stimulated macrophages produce large amounts of ROS, accompanied by NADPH oxidase activation, leading to a redox imbalance. Excess ROS diffuses to VSMCs and activates oxidative stress-responsive signaling pathways such as NF-κB and MAPK, prompting VSMCs to further release pro-inflammatory mediators and ROS, thereby establishing an oxidative stress–inflammation positive-feedback loop that exacerbates peroxidative damage in the vascular wall, promotes oxidative expansion of the lipid core, and disrupts the structural integrity of collagen within the fibrous cap [125]. Moreover, macrophage autophagic flux can indirectly modulate VSMC autophagy through the secretion of soluble factors. When macrophages exhibit autophagy deficiency, intracellular damaged mitochondria and oxidized lipids accumulate, resulting in reduced release of anti-inflammatory factors such as IL-10 and TGF-β and a substantially enhanced local pro-inflammatory microenvironment. This shift, in turn, suppresses or aberrantly activates VSMC autophagic flux, ultimately impairing VSMC metabolic status and proliferative capacity, increasing their susceptibility to apoptosis, and accelerating the disruption of vascular wall homeostasis [126].

VSMCs are not passive recipients of these regulatory signals; they reciprocally modulate macrophage recruitment, polarization, survival, and function through the same pathways—paracrine signaling, cell contact, exosomes, oxidative stress, and autophagy interactions—forming a complete bidirectional regulatory network. Under physiological conditions, VSMCs that maintain the contractile phenotype continuously secrete TGF-β and NO, which induce tissue-resident macrophages to retain an anti-inflammatory, pro-repair M2 polarization, thereby inhibiting excessive immune activation and maintaining vascular homeostasis, ultimately helping to reduce the local inflammatory burden and delay AS progression [127]. In response to vascular injury or inflammatory stress, VSMCs markedly upregulate MCP-1 expression, recruiting circulating monocytes into the vessel wall where they differentiate into activated macrophages, amplifying inflammatory signals, promoting foam cell formation, and exacerbating intra-plaque inflammatory cascades [128]. Meanwhile, VSMC-derived ECM components and surface adhesion molecules directly regulate macrophage adhesion, migration, and retention through contact-dependent mechanisms, further aggravating plaque inflammation and instability. Similar to macrophages, VSMCs also regulate macrophages in a contact-independent manner through exosome secretion, with bidirectional effects: under physiological conditions, exosomes from contractile-phenotype VSMCs carry anti-inflammatory miRNAs and homeostatic signals that induce M2 macrophage polarization; under pathological conditions, exosomes from synthetic-phenotype VSMCs undergo cargo reprogramming, carrying pro-inflammatory signals and lipid metabolic molecules that enhance M1 polarization and the foam cell formation capacity of macrophages. Activated VSMCs under oxidative stress generate lipid peroxides and ROS derivatives, which reciprocally stimulate macrophages to reinforce pro-inflammatory signals, promoting M1 polarization and inflammatory cytokine secretion, thus partnering with macrophages in an oxidative stress-positive-feedback loop that drives sustained plaque progression and increased vulnerability [129]. VSMC autophagy status can also reciprocally regulate macrophage lipid phagocytic efficiency and inflammatory secretion: impaired autophagy leads to the accumulation of misfolded proteins and lipids, releasing stress signals that enhance macrophage pro-inflammatory polarization and foam cell formation, whereas moderate autophagy maintains metabolic homeostasis and attenuates inflammatory stimuli. Autophagic imbalance between the two cell types exacerbates intra-plaque material accumulation, enlarges the necrotic core, and reduces plaque stability (Figure 5).

### 3.5. VSMCs and Lymphocytes

The progression of AS is accompanied by persistent vascular wall inflammation. Whereas traditional research has primarily focused on lipid deposition and foam cell formation, accumulating evidence now indicates that dynamic interactions between vascular wall cells and immune cells constitute the core driving force that propels AS from early fatty streaks to advanced vulnerable plaques. Within this complex cellular regulatory network, lymphocytes and VSMCs do not act independently; rather, they jointly contribute to sustained inflammatory amplification, aberrant phenotypic switching, pathological calcification, and vascular wall remodeling through paracrine signals, exosome-mediated communication, oxidative stress crosstalk, and autophagy regulation. Lymphocytes can initiate VSMC phenotypic switching and functional derangement via these pathways, while VSMCs can reciprocally amplify lymphocyte activation and inflammatory effects, thereby forming a progressively escalating pathological loop. The net effect of this bidirectional interaction is to jointly promote inflammatory infiltration, fibrous cap thinning, matrix degradation, and vascular wall stiffening, ultimately converting stable plaques into vulnerable plaques and increasing the risk of plaque rupture, thrombosis, and acute cardiovascular events.

In the atherosclerotic microenvironment, activated lymphocytes regulate VSMC function through multiple dimensions, including paracrine signaling, cell contact, exosomes, oxidative stress, and autophagy interference [130]. With regard to classical paracrine mechanisms, activated Th1 and CD8^+^ T cells secrete core pro-inflammatory cytokines such as IFN-γ and TNF-α, which directly act on VSMC surface receptors. IFN-γ binding activates the JAK-STAT1 pathway, leading to STAT1 phosphorylation and nuclear translocation, which inhibits contractile phenotype genes (α-SMA, SM22α) and upregulates synthetic phenotype genes (osteopontin, MMP9), thereby driving VSMC conversion toward a synthetic phenotype [131]. TNF-α binding to TNFR1 activates both NF-κB and MAPK pathways, regulating the transcription of pro-inflammatory cytokines as well as VSMC proliferation, migration, and apoptosis [132]. Beyond paracrine signaling, CD8^+^ T cells can exert cytotoxic effects through direct cell–cell contact by releasing perforin, granzyme B, and Fas ligand to induce VSMC apoptosis, directly weakening the structural stability of the fibrous cap [133]. Depletion of CD8^+^ T cells attenuates these effects, reducing aberrant VSMC differentiation and calcification [130]. On this basis, lymphocyte-derived exosomes serve as remote regulatory carriers that further enhance signal transmission. Exosomes from activated lymphocytes are enriched with miRNAs such as miR-155, miR-21, and miR-146a; among these, miR-155 targets and inhibits SOCS1, alleviating the negative regulation of the JAK-STAT pathway and amplifying the pro-inflammatory effect of IFN-γ, whereas miR-21 targets and inhibits PTEN, activating the PI3K/Akt pathway and significantly enhancing VSMC proliferation and migration [134]. Following uptake by VSMCs via endocytosis or membrane fusion, these exosomes enable long-distance and sustained signal regulation. Furthermore, lymphocytes activate VSMC NOX2/NOX4 NADPH oxidases via pro-inflammatory cytokines, inducing massive ROS production while suppressing endogenous antioxidant enzymes such as superoxide dismutase and glutathione peroxidase, leading to a redox imbalance [135]. The resulting ROS activates NF-κB, MAPK, and calcium channels through oxidative mechanisms, causing calcium overload and mitochondrial damage and forming a mitochondrial ROS–cytosolic ROS positive-feedback loop that persistently aggravates VSMC oxidative injury and dysfunction [136]. More importantly, lymphocyte-derived IFN-γ and TNF-α directly disrupt VSMC autophagy homeostasis. IFN-γ inhibits the formation of the ULK1 complex at the autophagy initiation stage via STAT1, while also suppressing autophagosome–lysosome fusion, leading to impaired autophagic flux. TNF-α upregulates Beclin-1 through NF-κB to promote autophagy initiation, yet inhibits lysosomal acidification and function, ultimately causing autophagosome accumulation. Impaired autophagic flux prevents VSMCs from clearing damaged mitochondria and oxidized proteins, resulting in toxic accumulation, oxidative stress, inflammatory amplification, and the promotion of apoptosis/necrosis with the release of DAMPs such as HMGB1, which further activates immune cells [137].

In the atherosclerotic microenvironment, VSMCs are not merely passive recipients of immune cell actions; they actively provide reciprocal regulation of lymphocytes through paracrine factor release, cell–cell contact, antigen presentation, and exosome-mediated information exchange. This bidirectional interaction between VSMCs and lymphocytes constitutes a complete regulatory network that profoundly participates in the maintenance and cascade amplification of chronic vascular inflammation. VSMCs primarily exert immunosuppressive effects by continuously secreting TGF-β and NO, thereby maintaining local immune balance and inhibiting excessive lymphocyte activation and proliferation [138]. However, under inflammatory stress, VSMCs undergo marked synthetic phenotypic switching and release large amounts of pro-inflammatory mediators and chemokines, notably CCL2 and CCL5 [139]. Simultaneously, under IFN-γ stimulation, VSMCs significantly upregulate MHC-II molecules and the co-stimulatory molecules CD80 and CD86, thereby converting into non-professional antigen-presenting cells capable of activating CD4^+^ T cells [140]. Additionally, CD40 on VSMCs binds CD40L on lymphocytes, bidirectionally activating NF-κB and MAPK pathways, promoting pro-inflammatory cytokine secretion, and establishing a positive-feedback loop. Exosomes also mediate this reverse regulatory axis, with their effects exhibiting high phenotype dependence. Exosomes released by contractile-phenotype VSMCs are enriched in smooth muscle-specific miRNAs such as miR-143 and miR-145, which effectively suppress lymphocyte activation and pro-inflammatory cytokine secretion [141]. Conversely, dedifferentiated synthetic-phenotype VSMCs release pathological exosomes enriched in pro-inflammatory miRNAs that synergize with free chemokines to directionally recruit peripheral lymphocytes into plaques and induce their pro-inflammatory polarization, thereby precisely reprogramming lymphocytes in terms of migration, activation, proliferation, and differentiation in a pathological manner [142]. Through these bidirectional interactions, VSMCs and lymphocytes form an intersecting and synergistic loop of inflammation and calcification: pro-inflammatory factors aggravate oxidative stress and calcium homeostasis imbalance, while functionally aberrant VSMCs further amplify inflammation, jointly driving lesion progression (Figure 6).

### 3.6. Macrophages and Lymphocytes

The interaction between macrophages and lymphocytes represents a core immunological event that regulates plaque development, establishing a complex and tightly orchestrated bidirectional regulatory network through antigen presentation, cytokine signaling, pathway transduction, and direct cell–cell contact. This network jointly contributes to inflammatory amplification, lipid metabolic dysregulation, and pathological plaque progression in AS, serving as a key regulatory mechanism throughout the disease course. Within this network, a pro-inflammatory positive-feedback loop centered on Th1/Th17 subsets and M1 macrophages continuously drives plaque inflammation, lipid deposition, and instability, while an anti-inflammatory negative-regulatory loop involving Tregs and macrophages maintains intra-plaque immune homeostasis. The dynamic balance between these two opposing forces may be critical in determining the pathological progression of AS.

Within this bidirectional network, macrophages first exert regulatory effects by mediating lymphocyte activation, differentiation, and function at multiple levels. As the major antigen-presenting cells (APCs) within plaques, macrophages take up ox-LDL via scavenger receptors (SR-A, CD36), process the protein components, such as ApoB-100, through lysosomal degradation into peptide antigens, and then load them onto MHC-II molecules for surface presentation. This peptide–MHC-II complex, together with T cell receptor (TCR) engagement and co-stimulatory signals provided by CD80/CD86 binding to CD28, initiates T-cell activation, proliferation, and differentiation [143]. Macrophages secrete IL-12 and other pro-inflammatory cytokines that drive T-cell differentiation toward the Th1 subset via the JAK-STAT pathway; in turn, Th1 cells secrete IFN-γ, which reciprocally enhances macrophage antigen presentation and pro-inflammatory functions, forming a positive regulatory loop. Concurrently, macrophages promote early T-cell differentiation toward the Th17 subset through IL-6, TGF-β, and IL-23, thereby enriching the functional repertoire of lymphocytes. Macrophage MHC-II expression is tightly controlled by the class II transactivator (CIITA); loss of MHC-II antigen-presenting capacity disrupts the immune activation loop and exacerbates atherosclerotic lesions in ApoE^−/−^ mice. At the level of membrane contact, CD40L on activated T cells binds CD40 on macrophages, activating NF-κB, MAPK, and other pro-inflammatory pathways, thereby promoting the secretion of pro-inflammatory cytokines, chemokines, and MMPs, enhancing ox-LDL uptake and foam cell formation. Activated macrophages, in turn, further upregulate CD40 and promote CD40L expression on lymphocytes, establishing a proinflammatory positive-feedback loop that drives plaque progression and destabilization [144,145]. Additionally, macrophages secrete extracellular vesicles carrying antigenic peptides, co-stimulatory molecules, and cytokines, which enable remote regulation of T-cell activation, differentiation, and function in a non-contact manner, thus contributing to transcellular immune signaling within plaques.

Lymphocytes, in turn, exert precise feedback regulation on macrophages through cytokine paracrine signaling, cytotoxic effects, membrane contact signals, and extracellular vesicle communication, thereby establishing a pro-inflammatory and anti-inflammatory bidirectional network with macrophages. Activated T-cell subsets secrete specific cytokines that directionally induce macrophage polarization: Th1 cells secrete IFN-γ and TNF-α, driving M1 pro-inflammatory polarization, promoting the release of TNF-α, IL-1β, and IL-6, and activating the NF-κB pathway to recruit immune cell infiltration [146]; certain cytotoxic T cells release perforin and granzymes to directly induce macrophage apoptosis, aggravating inflammatory dysregulation; Th17 cells secrete IL-17A, which upregulates macrophage SR expression, enhances ox-LDL uptake and foam cell formation, and activates NF-κB to promote pro-inflammatory cytokine release, thereby establishing a macrophage–Th17 pro-inflammatory positive-feedback loop. Tregs exert critical negative regulatory effects by secreting IL-10 and TGF-β, which inhibit M1 polarization, enhance efferocytosis, and reduce necrotic core formation, while also downregulating CD80 and CD86 expression via direct contact to weaken antigen-presenting capacity and prevent excessive T-cell activation. In turn, macrophages secrete anti-inflammatory factors that maintain Treg differentiation and functional stability, together constituting an intra-plaque negative immune regulatory loop [147,148]. Within this bidirectional network orchestrated by membrane contact and vesicular communication, Tregs can induce macrophage polarization toward a pro-resolving phenotype, inhibiting plaque progression and promoting regression; on the other hand, pro-inflammatory T cells potently stimulate macrophages to secrete large amounts of MMP-2 and MMP-9, which directly degrade vascular ECM, and synergistically promote VEGF release to induce intra-plaque neovascularization, ultimately exacerbating intra-plaque hemorrhage, lipid deposition, and adaptive immune infiltration, thereby driving plaques toward instability and vulnerability [149,150] (Figure 7).

### 3.7. Emerging Technologies and Concepts of Cell Crosstalk in Atherosclerosis

The preceding sections introduced the main cell types in atherosclerosis and their interactions, descriptions that were based largely on bulk-tissue and candidate-gene studies. Such work averages across cell states and overlooks where each cell actually sits in the vessel wall. In recent years, however, several new concepts have emerged, including single-cell and spatial omics, macrophage heterogeneity beyond the M1/M2 paradigm, and immune interactions involving neutrophils and platelets. These approaches offer a broader view of cell communication in atherosclerosis.

Single-cell RNA sequencing, single-cell ATAC-seq, CITE-seq and spatial transcriptomics allow the plaque to be analyzed cell by cell rather than as an average tissue sample. In the aortic endothelium, single-cell profiling has identified a hierarchy running from endovascular progenitor cells to differentiated endothelial cells [151]. In human lesions, endothelial cells fall into subsets that differ in angiogenic activity and show an endothelial-to-mesenchymal transition program, marked by loss of CLDN5 and CDH5 together with gain of mesenchymal markers, and these subsets can be placed at specific sites within the plaque [152]. Human aortic endothelial cells and lesion-derived endothelial cells also respond differently to IL-1beta and TGF-beta2, so inflammatory and matrix signals are not handled by a uniform endothelial layer [153]. Vascular smooth muscle cells are similarly diverse, as activated cells reduce ACTA2, MYH11 and CNN1 and become fibroblast-like fibromyocytes that produce COL1A1, DCN and LUM under TCF21, which in turn changes the matrix around endothelium and macrophages [154]. Spatial transcriptomics anchors these cell states to their actual locations and shows that plaque microvessels and adventitial endothelial cells act as signaling hubs that carry circulating signals into the wall [155]. Immune atlases add T lymphocytes and macrophages to this picture and reveal which cells lie next to one another and can therefore communicate [50]. In short, these methods turn isolated molecular events into a picture of how cells are arranged and how they exchange signals in the plaque.

Plaque macrophages were long described as either pro-inflammatory M1 or anti-inflammatory M2 cells, but this simple model does not explain how these cells interact with their neighbors [156]. Single-cell studies of the aorta instead find a continuum of states. In mouse lesions, these cluster into inflammatory cells expressing Il1b and Ccr2, resident-like cells, and TREM2-high foam cells rich in GPNMB and SPP1 [28]. These subsets occupy different regions and contact different cells rather than mixing evenly throughout the plaque [157]. The TREM2-high cells are specialized for lipid handling, since TREM2 binds lipoproteins and supports foam-cell lipid uptake and survival, and deleting Trem2 in macrophages reduces lesion growth and makes foam cells more likely to die [158]. Human carotid plaque atlases also place foam cells near the necrotic core and fibrous cap, next to smooth-muscle-derived matrix and apoptotic cells, rather than treating them as one uniform population [159]. As a direct example of such crosstalk, LYVE-1-positive resident macrophages interact with vascular smooth muscle cells through hyaluronan, showing that these macrophages exist within a smooth-muscle-built environment; treating atherosclerosis by simply switching macrophages from M1 to M2 would therefore disrupt an ongoing interaction among several cell types rather than resetting a single lineage [160].

Neutrophils and platelets also shape plaque crosstalk through direct contact and transferred molecules [13,161]. Neutrophils release neutrophil extracellular traps, which are decondensed chromatin coated with histones, elastase and myeloperoxidase. These traps catch nearby platelets and prompt macrophages to release IL-1beta and TNF-alpha, so a local response spreads through the plaque [162]. At sites of superficial erosion, the traps lie next to apoptotic endothelial cells, and TLR2-driven neutrophil activation adds to endothelial stress and detachment, linking innate immunity directly to endothelial injury [163]. Platelets complete this loop. Surface P-selectin binds PSGL-1 on neutrophils and monocytes, an interaction strengthened by GPIb and Mac-1, and platelets shed microvesicles carrying miR-126, miR-223 and miR-150 that enter nearby vascular and immune cells [164]. The resulting platelet-leukocyte aggregates and tissue-factor-bearing traps recruit and activate each other, forming a self-reinforcing loop among endothelium, neutrophils and platelets that extends beyond the usual structural cells [165].

## 4. Therapeutic Strategies Targeting Cellular Interactions

In recent years, beyond traditional lipid-lowering therapies, strategies targeting the interactions among various cell types within the vessel wall have emerged as a research hotspot. Several drugs have entered clinical studies or have been approved for the treatment of atherosclerotic cardiovascular disease. These agents exert their effects by interfering with the complex signaling crosstalk among ECs, smooth muscle cells, macrophages, and lymphocytes, thereby stabilizing plaques and slowing disease progression. From a mechanistic perspective, traditional lipid-lowering drugs have been shown to possess cell-interaction-modulating effects independent of lipid reduction; anti-inflammatory agents interrupt endothelial–smooth muscle crosstalk by inhibiting key inflammatory pathways; anticoagulants modulate macrophage–smooth muscle communication through coagulation factor Xa signaling; metabolic regulators affect immune cell lipid uptake and inflammatory responses; and novel biologics and small-molecule antagonists directly target bridging molecules involved in immune–vascular cell interactions (Table 1).

**Table 1:** Summary of Therapeutic Drugs Targeting Cell Interactions in Atherosclerosis.

Drug Name	Targeted Cell Interaction Type	Drug Category	Core Mechanism of Action		References
Colchicine	Endothelial Cell–Smooth Muscle Cell	Classic Anti-inflammatory Drug	Endothelial Cell	Inhibits endothelial cell senescence and SASP phenotype; reduces MMP-2 expression.	[166,167]
			Smooth Muscle Cell	Decreases the degradation of smooth muscle cell extracellular matrix by matrix metalloproteinases secreted by endothelial cells; blocks the pathological activation of smooth muscle cells by endothelial cells.	
Fluvastatin	Endothelial Cell–Smooth Muscle Cell	Statin Lipid-lowering Drug	Endothelial Cell	Reduces endothelial cell MMP-1 level.	[168,169,170]
			Smooth Muscle Cell	Reduces SMC extracellular-matrix degradation by endothelial MMPs; inhibits paracrine-induced SMC proliferation and inflammation; and downregulates CTGF/CCN2 to attenuate SMC proliferation, migration and ECM accumulation.	
HA-ML@ES NPs	Endothelial Cell–Macrophage	Dual-targeting Nanomedicine	Endothelial Cell	Blocks the endothelial cell NF-κB pathway; downregulates the expression of adhesion and chemotactic factors such as MCP-1, ICAM-1, and VCAM-1 secreted by endothelial cells.	[171]
			Macrophage	Cuts off the recruitment and inflammatory activation signals of macrophages by endothelial cells.	
Evolocumab	Endothelial Cell–Macrophage	PCSK9 Inhibitor	Endothelial Cell	Dampens PCSK9–CAP1/Syk/PKCδ-mediated macrophage inflammatory signaling.	[172,173]
			Macrophage	Cuts off the cascade positive-feedback loop composed of endothelial injury, monocyte recruitment, and macrophage inflammatory polarization.	
Atorvastatin, Pravastatin, Rosuvastatin	Endothelial Cell–Lymphocyte	Statin Lipid-lowering Drug	Endothelial Cell	Downregulates endothelial cell CX3CL1 expression; inhibits the CX3CL1-CX3CR1 signaling axis.	[174,175,176]
			Lymphocyte	Reduces the chemotactic recruitment and transendothelial migration of lymphocytes by endothelial cells.	
Metformin	Endothelial Cell–Lymphocyte	Hypoglycemic Drug	Macrophage	Downregulates the expression of macrophage scavenger receptors; reduces ox-LDL uptake.	[177,178]
			Lymphocyte	Indirectly blocks the lymphocyte inflammation amplification effect mediated by macrophages.	
Rivaroxaban	Smooth Muscle Cell–Macrophage	Oral Anticoagulant	Macrophage	Reduces the ability of macrophages to induce the transformation of smooth muscle cells from contractile phenotype to synthetic phenotype through Notch signaling.	[179]
			Smooth Muscle Cell	Inhibits the pathological phenotypic transformation of smooth muscle cells.	
Anti-CD40L Monoclonal Antibody	Smooth Muscle Cell–Lymphocyte	Biological Agent (Monoclonal Antibody)	Lymphocyte	Blocks the CD40L molecule on the surface of T lymphocytes; interrupts the CD40L-CD40 costimulatory pathway.	[180]
			Smooth Muscle Cell	Cuts off the pathogenic activation signal of smooth muscle cells by T lymphocytes.	
KAND567	Macrophage–Lymphocyte	Oral Small-Molecule CX3CR1 Antagonist	Macrophage	Blocks the CX3CL1/CX3CR1 signaling axis.	[181,182]
			Lymphocyte	Breaks the interaction activation loop between macrophages and T lymphocytes; weakens the T cell-driven pro-inflammatory amplification effect and macrophage M1 polarization.	

### 4.1. Anti-Inflammatory and Immunomodulatory Agents

Chronic inflammation constitutes the central driver of aberrant cellular crosstalk in atherosclerosis, fueling endothelial activation and senescence, macrophage pro-inflammatory polarization, and reciprocal activation between innate and adaptive immune cells within plaques. Anti-inflammatory and immunomodulatory agents interrupt these pathogenic signaling networks by targeting key inflammatory pathways, including NF-κB, NLRP3 inflammasome, and chemokine axes, thereby restoring homeostatic cell–cell communication and stabilizing vulnerable plaques.

Colchicine, a classic anti-inflammatory agent, has shown promise in the treatment of AS. Studies have reported that a novel colchicine-loaded nanodelivery system (VHPK-PLGA@COL) can specifically target inflamed ECs and enhance plaque stability by blocking the NF-κB/NLRP3 pathway, effectively alleviating plaque accumulation [166]. Additionally, colchicine modulates EC-VSMC interaction through the regulation of MMPs. It inhibits the NF-κB, MAPK (p38/ERK), and AKT/mTOR pathways, thereby reducing oxidative stress-induced endothelial senescence and SASP, significantly decreasing MMP-2 expression, and increasing TIMP levels, which together reduce ECM degradation, enhance plaque stability, and exert vascular protective effects [167].

KAND567 is a selective oral small-molecule CX3CR1 antagonist currently under clinical investigation. Within atherosclerotic plaques, macrophages and T lymphocytes establish a positive-feedback activation loop, macrophages secrete the chemokine CX3CL1, which recruits and activates T cells expressing its receptor CX3CR1, while activated T cells in turn promote M1 macrophage polarization, thereby amplifying inflammation. By blocking the CX3CL1/CX3CR1 axis, KAND567 rapidly and reversibly suppresses the recruitment and activation of CX3CR1-expressing lymphocytes, particularly effector T cells, to inflammatory sites, thereby limiting their infiltration into the vessel wall; it also disrupts the reciprocal activation between plaque macrophages and T lymphocytes, attenuates T-cell-driven pro-inflammatory amplification and M1 polarization, and curtails the inflammatory cascade within the vascular wall. Through this dual action, oral KAND567 reduces macrophage infiltration into the vessel wall, alleviates the local inflammatory burden, and delays lesion progression [181], ultimately slowing atherosclerotic plaque progression while enhancing plaque stability, and achieving precise intervention in atherosclerosis at the level of adaptive immunity [182].

### 4.2. Lipid-Lowering Agents with Pleiotropic Vascular Effects

Beyond their canonical lipid-lowering effects, statins and proprotein convertase subtilisin/kexin type 9 (PCSK9) inhibitors exert pleiotropic vascular-protective actions that directly modulate intercellular crosstalk among endothelial cells, vascular smooth muscle cells, macrophages, and lymphocytes. These agents reduce endothelial adhesion molecule and chemokine expression, inhibit VSMC phenotypic switching and proliferation, suppress macrophage foam cell formation, and limit lymphocyte transendothelial migration, collectively interrupting the inflammatory cascades that drive plaque progression independent of lipid reduction.

Fluvastatin reduces basal MMP-1 levels in human EC culture medium, enhancing plaque stability and effectively slowing the progression of AS [168]. Studies have shown that fluvastatin effectively reduces cholesterol content in VSMCs, potentially inhibiting VSMC migration during AS progression [169]. Furthermore, fluvastatin treatment significantly reduces CTGF (also known as CCN2) mRNA and protein levels in VSMCs, thereby attenuating VSMC proliferation, migration, and extracellular matrix accumulation [183]. These effects ultimately attenuate endothelial dysfunction, inhibit aberrant VSMC proliferation, reduce intimal hyperplasia and smooth muscle cell accumulation within plaques, lower circulating pro-inflammatory cytokine levels, and alleviate AS.

Studies have shown that treatment with atorvastatin, pravastatin, and rosuvastatin significantly decreases fractalkine CX3CL1 levels, suggesting that statins may exert anti-inflammatory effects beyond lipid-lowering by downregulating CX3CL1 expression and reducing lymphocyte migration and adhesion to the vessel wall [174]. In parallel, atorvastatin inhibits endothelial ferroptosis by modulating intracellular iron content, MDA levels, ROS levels, and ACSL4 protein expression, thereby reducing vascular EC damage and death [175]. Moreover, by lowering CX3CL1 levels and inhibiting the CX3CL1–CX3CR1 signaling axis, atorvastatin effectively reduces immune cell transendothelial migration and infiltration into arterial inflammatory sites, alleviates endothelial inflammatory injury, suppresses intra-plaque inflammatory responses and foam cell formation, and ultimately exerts non-lipid-lowering anti-atherosclerotic effects such as stabilizing plaques and delaying lesion progression [176].

Evolocumab, a representative PCSK9 inhibitor of the monoclonal antibody class, significantly increases the survival rate of human umbilical vein endothelial cells (HUVECs), exerting endothelium-protective functions independent of its lipid-lowering effects [172]. More importantly, PCSK9 itself promotes inflammation and accelerates atherosclerosis independently of the LDL receptor, engaging CAP1 as its major binding partner and signaling through Syk/PKCδ; notably, a CAP1-Fc fusion protein blocks PCSK9-driven inflammatory signaling more effectively than evolocumab, which neutralizes PCSK9 only for LDLR degradation and does not disrupt the PCSK9–CAP1 interaction [173].

### 4.3. Anticoagulant, Metabolic, and Co-Stimulatory Targeting Agents

Emerging evidence supports the therapeutic potential of agents originally developed for non-atherosclerotic indications, including anticoagulants, metabolic regulators, and immune co-stimulatory pathway blockers, in modulating pathogenic cellular interactions within atherosclerotic lesions. These agents act through distinct mechanistic axes, namely coagulation factor signaling, metabolic and scavenger receptor pathways, and T-cell co-stimulatory molecules, to disrupt pro-atherogenic communication between macrophages and VSMCs, immune cells and VSMCs, and other key cellular pairs.

Rivaroxaban, an oral anticoagulant, is a representative agent of recent drug-repurposing strategies targeting the interaction between macrophages and VSMCs, a key driver of neointima formation and plaque progression. Beyond its anticoagulant effects, rivaroxaban reduces macrophage pro-inflammatory polarization and Notch ligand expression by blocking relevant receptor signaling on macrophages. Mechanistically, rivaroxaban inhibits activated coagulation factor X (FXa), thereby blocking the FXa-induced PAR2/Akt/HIF1α signaling pathway and downregulating M1 polarization and Dll4 overexpression in macrophages. Given that M1 macrophages drive the Notch-mediated conversion of VSMCs from a contractile to a synthetic phenotype, which confers proliferative, migratory, and matrix-secreting capacities and exacerbates luminal narrowing, the resulting reduction in M1 macrophage numbers and Dll4 expression diminishes their capacity to induce VSMC phenotypic switching. Consequently, the aberrant contractile-to-synthetic transition of VSMCs is curbed, ultimately slowing atherosclerotic plaque formation and progression [179]. Moreover, rivaroxaban inhibits the inflammatory activation of both macrophages and VSMCs and attenuates neointima formation following wire-induced vascular injury.

Metformin has been shown to have potential in suppressing endothelial pro-inflammatory responses and can inhibit macrophage lipid uptake [177], thereby curbing AS progression. In atherosclerotic lesions, macrophage uptake of ox-LDL and subsequent apoptosis are key steps in plaque deterioration. Metformin downregulates the expression of scavenger receptors SR-A and CD36 by inhibiting the β-catenin, AP-1, and PPAR-γ pathways, significantly reducing ox-LDL uptake. Simultaneously, it inhibits ox-LDL-induced ER stress, ROS production, mitochondrial membrane depolarization, and pro-inflammatory cytokine release, thereby blocking the inflammatory cascade within the vessel wall and exerting potent anti-atherosclerotic effects [178].

Anti-CD40L antibodies represent a specific therapeutic strategy targeting the CD40L-CD40 co-stimulatory pathway, a key node in the imbalance between plaque immune inflammation and fibrosis that arises from bidirectional activation between intimal T lymphocytes and VSMCs. In this axis, CD40L on activated T cells binds CD40 on VSMCs, prompting VSMCs to secrete MMPs and reduce collagen synthesis, thereby leading to fibrous cap thinning and plaque destabilization. By binding to and blocking CD40L on T cells, anti-CD40L antibodies interrupt the pathogenic activation signal to VSMCs at its source, thereby inhibiting MMP secretion and restoring and enhancing collagen synthesis. Studies have confirmed that inhibiting CD40L-CD40 signaling increases intra-plaque collagen content, elevates the VSMC myofibroblast ratio, and thickens the fibrous cap, converting vulnerable atherosclerotic lesions into stable ones. This approach thereby fundamentally blocks immune cell-mediated plaque destabilization, slows AS progression, and reduces the incidence of acute cardiovascular events [180].

### 4.4. Nanoparticle-Based Dual-Targeting Therapeutic Systems

Nanoparticle-based drug delivery systems represent an innovative therapeutic paradigm that enables simultaneous targeting of multiple cell types and pathogenic pathways within the atherosclerotic microenvironment. By engineering dual-targeting nanocarriers with cell-specific ligands, these systems can concurrently protect endothelial cells, reverse macrophage pro-inflammatory polarization, and restore cholesterol efflux homeostasis, thereby interrupting cellular crosstalk at multiple nodes with enhanced precision and reduced off-target effects.

HA-ML@ES NPs are dual-targeting nanodrugs. Studies have shown that HA-ML@ES NPs inhibit EC apoptosis, reverse M1 macrophage polarization, eliminate inflammation, and suppress foam cell formation, ultimately facilitating the restoration of cholesterol flux homeostasis and intervening in AS progression. Moreover, HA-ML@ES NPs restore cholesterol efflux homeostasis by inhibiting the LOX-1-mediated endocytic pathway, upregulating ABCG1 expression, and suppressing endothelial NF-κB pathway activation, which results in significant downregulation of EC-secreted MCP-1, ICAM-1, VCAM-1, inflammatory cytokines, and ROS—key bridging factors between ECs and macrophages. By interrupting the inflammatory positive-feedback loop and attenuating damage signal crosstalk, these nanoparticles block the AS cascade at the level of cellular communication, thereby alleviating the progression of AS [171].

## 5. Conclusions and Perspectives

Atherosclerosis is a chronic progressive inflammatory disease of the vessel wall, whose pathological evolution involves the interplay of multiple mechanisms, including endothelial dysfunction, lipid deposition, intimal hyperplasia, and chronic inflammation, with the coordinated involvement of various cell types. This review systematically summarizes the core regulatory roles and molecular mechanisms of intercellular communication in the initiation and progression of atherosclerosis. Studies have shown that the major cellular components within plaques do not act in isolation; instead, they form an integrated intercellular communication network through paracrine signaling, exosome transfer, direct cell–cell contact, and oxidative stress–autophagy crosstalk. Signaling axes mediated by TGF-β/SMAD, Notch, and non-coding RNAs coordinate endothelial–VSMC interactions; macrophages and VSMCs establish positive-feedback loops via PDGF, MCP-1, and exosomal miRNAs; and macrophage–lymphocyte crosstalk is regulated by antigen presentation, CD40-CD40L, and PD-1/PD-L1 immune checkpoints, with the dynamic balance of these interactions ultimately determining plaque fate. Intervention strategies targeting key nodes of intercellular communication have demonstrated promising efficacy in animal models, inhibiting plaque progression, stabilizing vulnerable plaques, and attenuating vascular inflammatory injury, thereby providing potential targets and experimental evidence for the prevention and treatment of atherosclerosis.

Despite emerging insights into plaque cellular heterogeneity and localized signaling pathways, the translation of these findings from basic research to clinical application remains in its infancy. Existing studies rely heavily on animal models and are generally centered on isolated pathways, leaving a paucity of translational evidence required for clinical risk stratification. Although recent spatial-omic and functional studies, ranging from mapping senescent cell niches to delineating macrophage transdifferentiation trajectories and elucidating the mechanisms of efferocytosis, have provided important research foundations, these efforts remain largely piecemeal and have not yet formed a systematic research framework [184,185,186,187,188]. Moving forward, future studies should further delineate the molecular mechanisms underlying intercellular crosstalk in atherosclerosis and strengthen human-centric, multi-omic, and spatially resolved systems biology research to systematically decipher dynamic cellular networks within human lesions. Promoting the translational application of relevant therapeutic targets and developing novel therapeutic agents and strategies will open new avenues for the precise diagnosis and effective treatment of atherosclerosis.

## Figures and Tables

**Figure 1:**
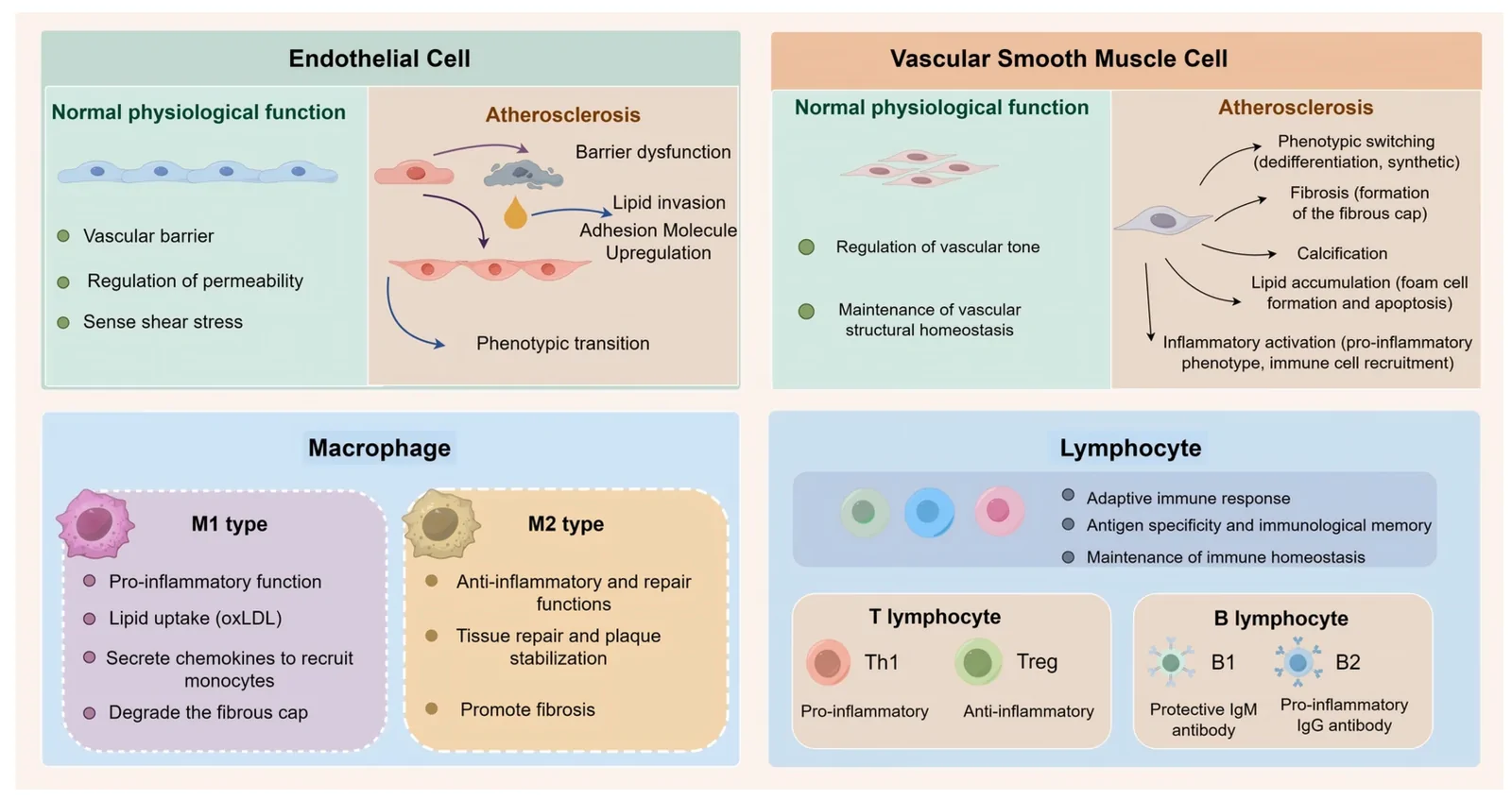
Cellular components and principal functions in atherosclerotic lesions. ECs exhibit barrier dysfunction, lipid invasion, adhesion molecule upregulation, and phenotypic transition; VSMCs undergo phenotypic switching, fibrosis, calcification, lipid accumulation, foam cell formation, and inflammatory activation. Macrophages are classified into pro-inflammatory M1 and anti-inflammatory M2 subsets; the M1 subset takes up oxidized LDL, secretes chemokines, and degrades the fibrous cap. Lymphocytes comprise Th1/Treg T cells and B1/B2 cells exerting opposing pro- and anti-inflammatory effects. This figure was created by FigDraw.com.

**Figure 2:**
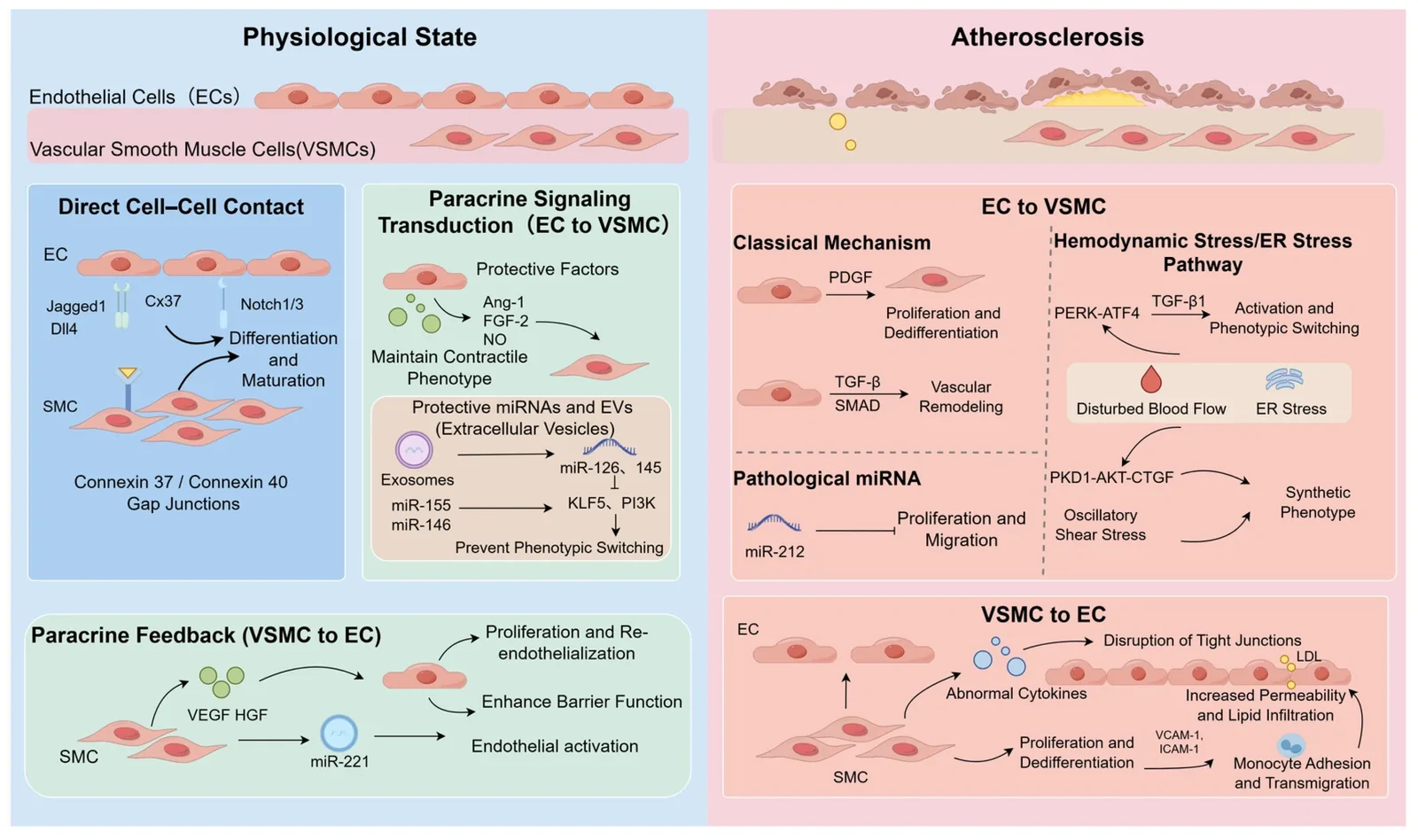
Bidirectional regulatory network between endothelial cell and vascular smooth muscle cell in atherosclerosis. In healthy vessels, direct cell–cell contact and paracrine signaling via Jagged1/Dll4-Notch, gap junctions, and protective factors and microRNAs maintain EC–VSMC homeostasis, including the contractile phenotype, barrier integrity, and endothelial regeneration. In atherosclerosis, disturbed hemodynamics and ER stress activate PDGF, TGF-β/SMAD, and pathological miRNA signaling, driving EC and VSMC dedifferentiation, proliferation, phenotypic switching, and vascular remodeling, whereas VSMC-derived signals disrupt tight junctions, increase permeability, and promote lipid infiltration and monocyte transmigration. This figure was created by FigDraw.com.

**Figure 3:**
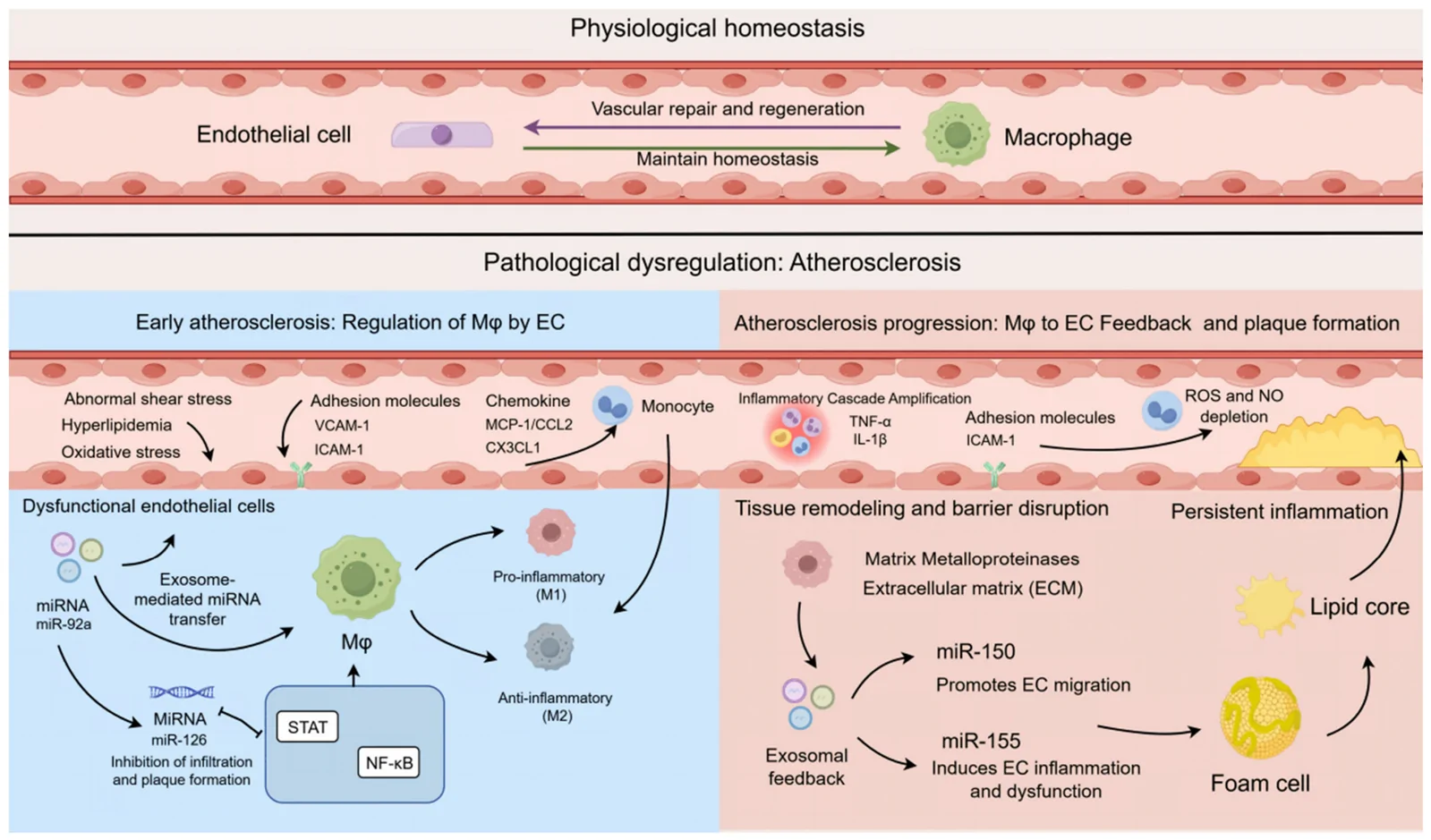
Bidirectional regulatory network between ECs and macrophages in atherosclerosis. EC–Mφ interactions sustain vascular repair, regeneration, and homeostasis. In early atherosclerosis, disturbed shear stress, hyperlipidemia, and oxidative stress promote EC dysfunction and monocyte recruitment via adhesion molecules (VCAM-1, ICAM-1) and chemokines (MCP-1/CCL2, CX3CL1). EC-derived exosomal microRNAs (miR-92a, miR-126) regulate Mφ STAT/NF-κB signaling, skewing toward pro-inflammatory M1 or anti-inflammatory M2 polarization. During progression, inflammatory cascades (TNF-α, IL-1β), MMP-mediated ECM remodeling, and exosomal feedback (miR-150, miR-155) cause foam cell and lipid core formation, barrier disruption, and persistent inflammation, promoting plaque formation. This figure was created by FigDraw.com.

**Figure 4:**
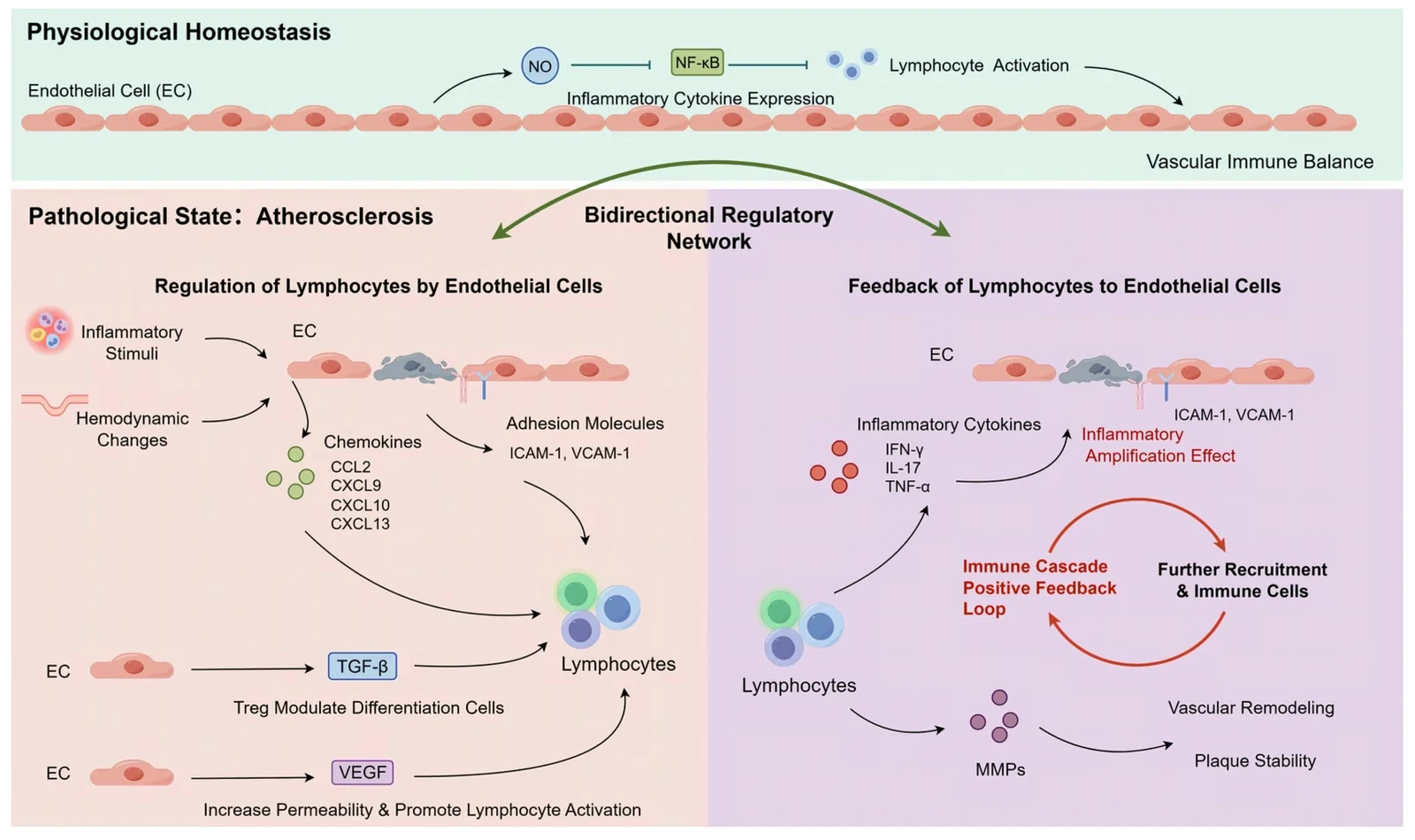
Bidirectional regulatory network between endothelial cells and lymphocytes in atherosclerosis. Under physiological conditions, EC-derived NO modulates NF-κB-dependent inflammatory cytokine expression and lymphocyte activation, maintaining vascular immune balance. In atherosclerosis, inflammatory stimuli and hemodynamic changes induce EC dysfunction, which recruits and activates lymphocytes via chemokines (CCL2, CXCL9, CXCL10, CXCL13), adhesion molecules (ICAM-1, VCAM-1), and EC-derived TGF-β and VEGF. In turn, lymphocyte-derived inflammatory cytokines (IFN-γ, IL-17, TNF-α) amplify EC inflammation, forming a positive-feedback loop that further recruits immune cells, whereas lymphocyte-derived MMPs mediate vascular remodeling and affect plaque stability. This figure was created by FigDraw.com.

**Figure 5:**
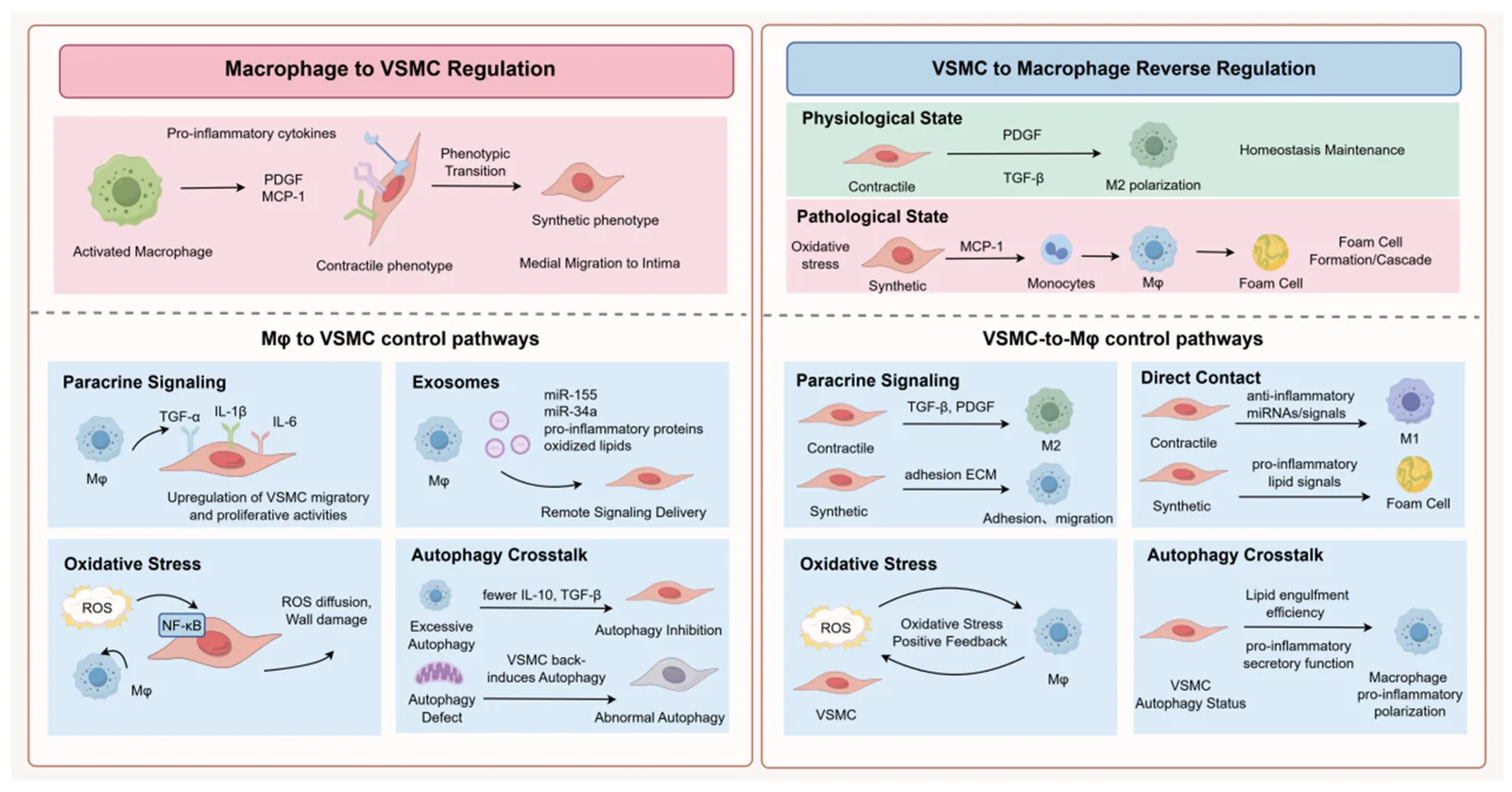
Bidirectional regulatory network between vascular smooth muscle cells and macrophages in atherosclerosis. Activated macrophages secrete PDGF and MCP-1 to drive VSMC phenotypic switching from contractile to synthetic states and intimal migration, via paracrine cytokines (TGF-α, IL-1β, IL-6), exosomal miR-155/miR-34a, ROS/NF-κB-mediated oxidative stress, and autophagy crosstalk. Conversely, contractile VSMCs promote M2 polarization and homeostasis through PDGF and TGF-β, whereas synthetic VSMCs recruit monocytes via MCP-1, enhance macrophage adhesion and migration, and, through direct contact, oxidative stress, and autophagy modulation, promote foam cell formation and pro-inflammatory polarization. This figure was created by FigDraw.com.

**Figure 6:**
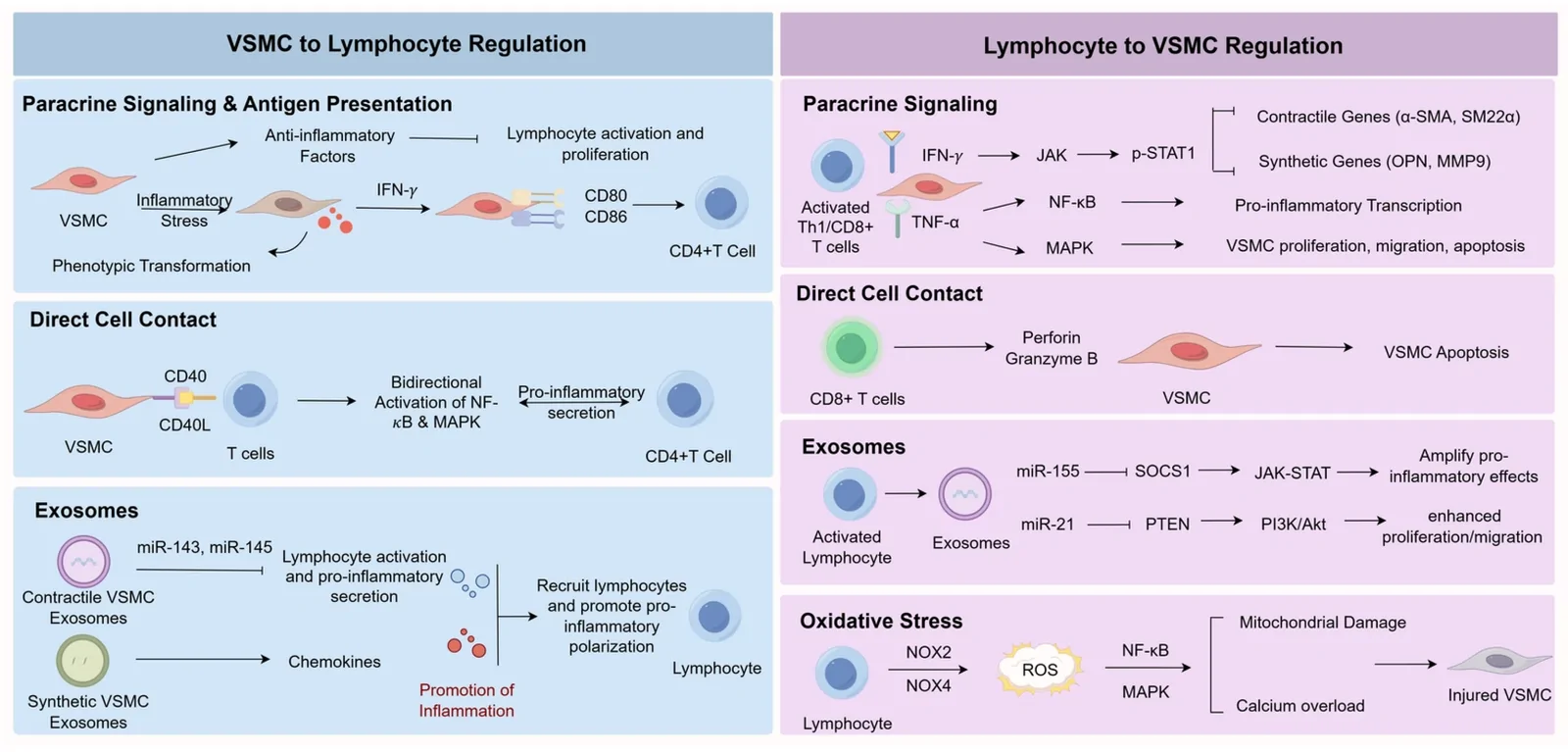
Bidirectional regulatory network between vascular smooth muscle cells and lymphocytes in atherosclerosis. VSMCs regulate lymphocytes through paracrine signaling and antigen presentation (anti-inflammatory factors, IFN-γ, CD80/CD86), CD40–CD40L-mediated direct contact activating NF-κB/MAPK, and exosomal miR-143/miR-145 or chemokines that promote lymphocyte activation and pro-inflammatory polarization. Conversely, activated Th1/CD8+ T cells drive VSMC phenotypic switching, proliferation, migration, and apoptosis via IFN-γ/JAK-STAT, TNF-α/NF-κB/MAPK, perforin/granzyme B-mediated cytotoxicity, exosomal miR-155/miR-21, and NOX2/NOX4-derived ROS. This figure was created by FigDraw.com.

**Figure 7:**
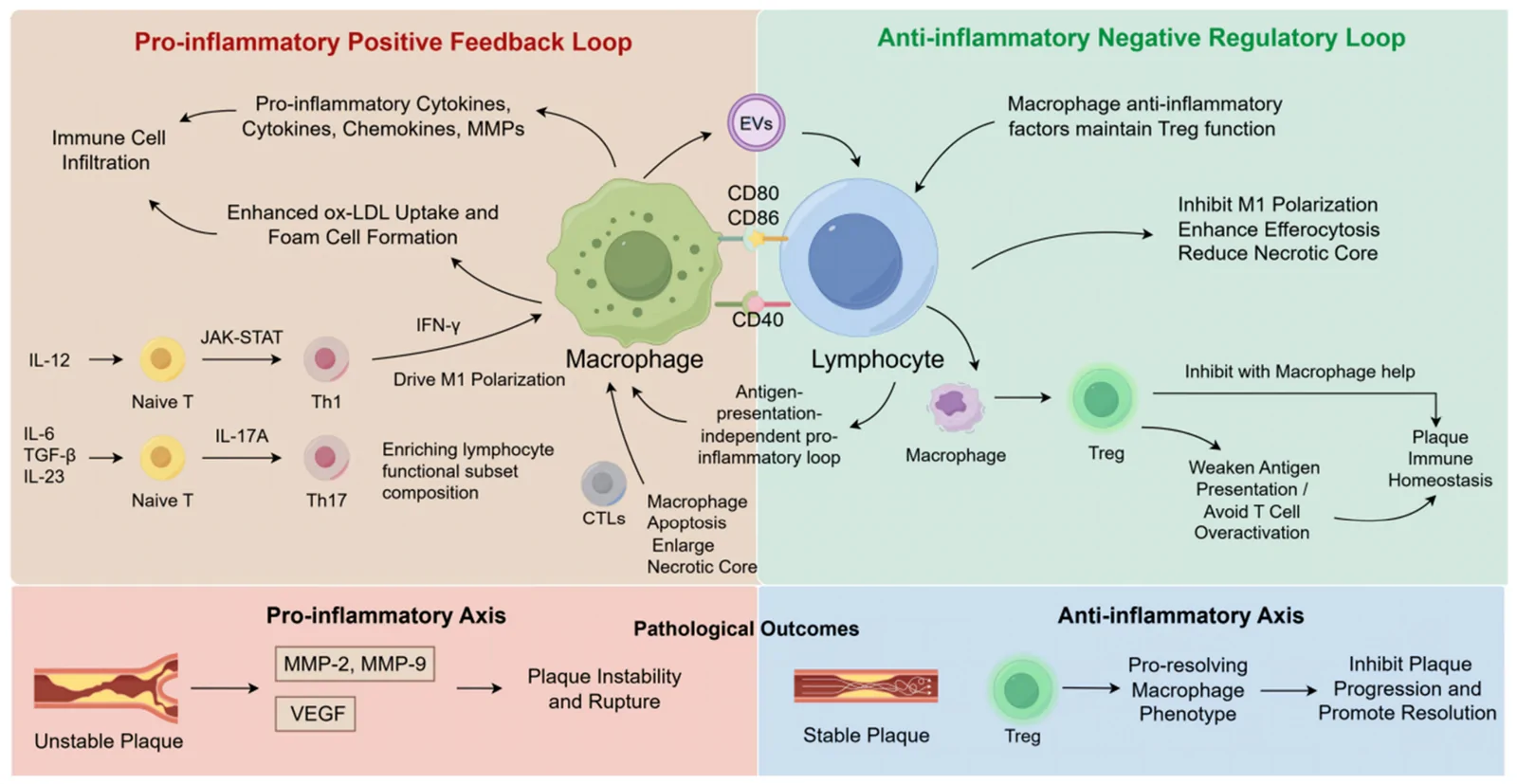
Bidirectional regulatory network between macrophages and lymphocytes in atherosclerosis. Pro-inflammatory feedback, mediated by CD80/CD86 and CD40, drives macrophage secretion of cytokines, chemokines, and MMPs, promoting immune infiltration, ox-LDL uptake, and foam cell formation; IFN-γ and Th1/Th17 polarization reinforce M1 activation and CTL-mediated apoptosis, enlarging the necrotic core and destabilizing plaques via MMP-2/MMP-9 and VEGF. Conversely, anti-inflammatory negative regulation relies on macrophage-derived factors that maintain Treg function, inhibit M1 polarization, enhance efferocytosis, and promote pro-resolving macrophage phenotype, suppressing plaque progression and restoring immune homeostasis. This figure was created by FigDraw.com.

## Data Availability

No datasets were generated or analysed during the current study.

## Abbreviations

The following abbreviations are used in this manuscript:

Abbreviation	Full Form
AS	Atherosclerosis
EC	Endothelial cell
VSMC	Vascular smooth muscle cell
Mφ	Macrophage
LDL	Low-density lipoprotein
ox-LDL	Oxidized low-density lipoprotein
NO	Nitric oxide
ECM	Extracellular matrix
MMP	Matrix metalloproteinase
ROS	Reactive oxygen species
NF-κB	Nuclear factor kappa-light-chain-enhancer of activated B cells
TNF-α	Tumor necrosis factor-alpha
IL	Interleukin
IFN-γ	Interferon-gamma
TGF-β	Transforming growth factor-beta
Treg	Regulatory T cell
Th	T helper
EndMT	Endothelial-to-mesenchymal transition
SR	Scavenger receptor
CD36	Cluster of differentiation 36
PCSK9	Proprotein convertase subtilisin/kexin type 9
CX3CL1/CX3CR1	Fractalkine/fractalkine receptor
PDGF	Platelet-derived growth factor
MCP-1	Monocyte chemoattractant protein-1
ICAM-1	Intercellular adhesion molecule-1
VCAM-1	Vascular cell adhesion molecule-1
MHC	Major histocompatibility complex
APC	Antigen-presenting cell
TCR	T cell receptor
JNK	c-Jun N-terminal kinase
STAT	Signal transducer and activator of transcription
CD40L	CD40 ligand
HUVEC	Human umbilical vein endothelial cell
DAMP	Damage-associated molecular pattern
NET	Neutrophil extracellular trap
ATLO	Artery tertiary lymphoid organ

## References

[1] Xing Y., Lin X. (2025). Challenges and advances in the management of inflammation in atherosclerosis. J. Adv. Res..

[2] Neels J.G., Gollentz C., Chinetti G. (2023). Macrophage death in atherosclerosis: Potential role in calcification. Front. Immunol..

[3] Xiang Q., Tian F., Xu J., Du X., Zhang S., Liu L. (2022). New insight into dyslipidemia-induced cellular senescence in atherosclerosis. Biol. Rev. Camb. Philos. Soc..

[4] Ajoolabady A., Pratico D., Lin L., Mantzoros C.S., Bahijri S., Tuomilehto J., Ren J. (2024). Inflammation in atherosclerosis: Pathophysiology and mechanisms. Cell Death Dis..

[5] Schlereth K., Weichenhan D., Bauer T., Heumann T., Giannakouri E., Lipka D., Jaeger S., Schlesner M., Aloy P., Eils R. (2018). The transcriptomic and epigenetic map of vascular quiescence in the continuous lung endothelium. eLife.

[6] Bjorkegren J.L.M., Lusis A.J. (2022). Atherosclerosis: Recent developments. Cell.

[7] Kwak B.R., Back M., Bochaton-Piallat M.L., Caligiuri G., Daemen M.J., Davies P.F., Hoefer I.E., Holvoet P., Jo H., Krams R. (2014). Biomechanical factors in atherosclerosis: Mechanisms and clinical implications. Eur. Heart J..

[8] Orsenigo F., Giampietro C., Ferrari A., Corada M., Galaup A., Sigismund S., Ristagno G., Maddaluno L., Koh G.Y., Franco D. (2012). Phosphorylation of VE-cadherin is modulated by haemodynamic forces and contributes to the regulation of vascular permeability in vivo. Nat. Commun..

[9] Libby P., Soehnlein O. (2025). Inflammation in atherosclerosis: Lessons and therapeutic implications. Immunity.

[10] Dong Y., Wang B., Du M., Zhu B., Cui K., Li K., Yuan K., Cowan D.B., Bhattacharjee S., Wong S. (2023). Targeting Epsins to Inhibit Fibroblast Growth Factor Signaling While Potentiating Transforming Growth Factor-beta Signaling Constrains Endothelial-to-Mesenchymal Transition in Atherosclerosis. Circulation.

[11] Chen P.Y., Qin L., Baeyens N., Li G., Afolabi T., Budatha M., Tellides G., Schwartz M.A., Simons M. (2015). Endothelial-to-mesenchymal transition drives atherosclerosis progression. J. Clin. Investig..

[12] Evrard S.M., Lecce L., Michelis K.C., Nomura-Kitabayashi A., Pandey G., Purushothaman K.R., d’Escamard V., Li J.R., Hadri L., Fujitani K. (2016). Endothelial to mesenchymal transition is common in atherosclerotic lesions and is associated with plaque instability. Nat. Commun..

[13] Alvandi Z., Bischoff J. (2021). Endothelial-Mesenchymal Transition in Cardiovascular Disease. Arterioscler. Thromb. Vasc. Biol..

[14] Zhai M., Lei Z., Shi Y., Shi J., Zeng Y., Gong S., Jian W., Zhuang J., Yu Q., Feinberg M.W. (2025). TEAD1-Mediated Trans-Differentiation of Vascular Smooth Muscle Cells into Fibroblast-Like Cells Contributes to the Stabilization and Repair of Disrupted Atherosclerotic Plaques. Adv. Sci..

[15] Luo L., Fu C., Bell C.F., Wang Y., Leeper N.J. (2023). Role of vascular smooth muscle cell clonality in atherosclerosis. Front. Cardiovasc. Med..

[16] Wang Y.T., Moura A.K., Zuo R., Gao W., McConnell B.K., Li G., Li P.L., Li X., Zhang Y. (2026). Vascular smooth muscle-specific NLRP3 hyperactivation exacerbates arterial intimal hyperplasia in mice. J. Mol. Med..

[17] Serbulea V., Martin J.M., Owens G.K. (2026). Role of Diverse Smooth Muscle Cell Phenotypic Transitions in Atherosclerosis Development and Late-Stage Pathogenesis. Annu. Rev. Physiol..

[18] Li M., Zhu Y., Jaiswal S.K., Liu N.F. (2021). Mitochondria Homeostasis and Vascular Medial Calcification. Calcif. Tissue Int..

[19] Allahverdian S., Chehroudi A.C., McManus B.M., Abraham T., Francis G.A. (2014). Contribution of intimal smooth muscle cells to cholesterol accumulation and macrophage-like cells in human atherosclerosis. Circulation.

[20] Wang Y., Dubland J.A., Allahverdian S., Asonye E., Sahin B., Jaw J.E., Sin D.D., Seidman M.A., Leeper N.J., Francis G.A. (2019). Smooth Muscle Cells Contribute the Majority of Foam Cells in ApoE (Apolipoprotein E)-Deficient Mouse Atherosclerosis. Arterioscler. Thromb. Vasc. Biol..

[21] Boyle J.J., Bowyer D.E., Weissberg P.L., Bennett M.R. (2001). Human blood-derived macrophages induce apoptosis in human plaque-derived vascular smooth muscle cells by Fas-ligand/Fas interactions. Arterioscler. Thromb. Vasc. Biol..

[22] Yin Z., Zhang J., Shen Z., Qin J.J., Wan J., Wang M. (2024). Regulated vascular smooth muscle cell death in vascular diseases. Cell Prolif..

[23] Grootaert M.O.J., Moulis M., Roth L., Martinet W., Vindis C., Bennett M.R., De Meyer G.R.Y. (2018). Vascular smooth muscle cell death, autophagy and senescence in atherosclerosis. Cardiovasc. Res..

[24] Yu Y., Cai Y., Yang F., Yang Y., Cui Z., Shi D., Bai R. (2024). Vascular smooth muscle cell phenotypic switching in atherosclerosis. Heliyon.

[25] Xu C., Ke C., Zhong Y.L., Yang X.H., Wang M.M., Cao Y., Chen J. (2026). Macrophage senescence and programmed cell death in atherosclerosis: Mechanisms, cross-talk, and emerging therapeutic strategies. Ageing Res. Rev..

[26] Thayaparan D., Emoto T., Khan A.B., Besla R., Hamidzada H., El-Maklizi M., Sivasubramaniyam T., Vohra S., Hagerman A., Nejat S. (2025). Endothelial dysfunction drives atherosclerotic plaque macrophage-dependent abdominal aortic aneurysm formation. Nat. Immunol..

[27] Erbel C., Tyka M., Helmes C.M., Akhavanpoor M., Rupp G., Domschke G., Linden F., Wolf A., Doesch A., Lasitschka F. (2015). CXCL4-induced plaque macrophages can be specifically identified by co-expression of MMP7+S100A8+ in vitro and in vivo. Innate Immun..

[28] Cochain C., Vafadarnejad E., Arampatzi P., Pelisek J., Winkels H., Ley K., Wolf D., Saliba A.E., Zernecke A. (2018). Single-Cell RNA-Seq Reveals the Transcriptional Landscape and Heterogeneity of Aortic Macrophages in Murine Atherosclerosis. Circ. Res..

[29] Newby A.C., George S.J., Ismail Y., Johnson J.L., Sala-Newby G.B., Thomas A.C. (2009). Vulnerable atherosclerotic plaque metalloproteinases and foam cell phenotypes. Thromb. Haemost..

[30] Park Y.M., Febbraio M., Silverstein R.L. (2009). CD36 modulates migration of mouse and human macrophages in response to oxidized LDL and may contribute to macrophage trapping in the arterial intima. J. Clin. Investig..

[31] Park Y.M. (2014). CD36, a scavenger receptor implicated in atherosclerosis. Exp. Mol. Med..

[32] Hirose K., Iwabuchi K., Shimada K., Kiyanagi T., Iwahara C., Nakayama H., Daida H. (2011). Different responses to oxidized low-density lipoproteins in human polarized macrophages. Lipids Health Dis..

[33] Tannahill G.M., Curtis A.M., Adamik J., Palsson-McDermott E.M., McGettrick A.F., Goel G., Frezza C., Bernard N.J., Kelly B., Foley N.H. (2013). Succinate is an inflammatory signal that induces IL-1beta through HIF-1alpha. Nature.

[34] Saha S., Shalova I.N., Biswas S.K. (2017). Metabolic regulation of macrophage phenotype and function. Immunol. Rev..

[35] Winter C., Silvestre-Roig C., Ortega-Gomez A., Lemnitzer P., Poelman H., Schumski A., Winter J., Drechsler M., de Jong R., Immler R. (2018). Chrono-pharmacological Targeting of the CCL2-CCR2 Axis Ameliorates Atherosclerosis. Cell Metab..

[36] Tabas I., Bornfeldt K.E. (2016). Macrophage Phenotype and Function in Different Stages of Atherosclerosis. Circ. Res..

[37] Newby A.C. (2008). Metalloproteinase expression in monocytes and macrophages and its relationship to atherosclerotic plaque instability. Arterioscler. Thromb. Vasc. Biol..

[38] Johnson J.L., Jenkins N.P., Huang W.C., Di Gregoli K., Sala-Newby G.B., Scholtes V.P., Moll F.L., Pasterkamp G., Newby A.C. (2014). Relationship of MMP-14 and TIMP-3 expression with macrophage activation and human atherosclerotic plaque vulnerability. Mediat. Inflamm..

[39] Gu C., Wang F., Zhao Z., Wang H., Cong X., Chen X. (2017). Lysophosphatidic Acid Is Associated with Atherosclerotic Plaque Instability by Regulating NF-kappaB Dependent Matrix Metalloproteinase-9 Expression via LPA(2) in Macrophages. Front. Physiol..

[40] Wu N.Q., Shi H.W., Li J.J. (2022). Proprotein Convertase Subtilisin/Kexin Type 9 and Inflammation: An Updated Review. Front. Cardiovasc. Med..

[41] van Dierendonck X., Vrieling F., Smeehuijzen L., Deng L., Boogaard J.P., Croes C.A., Temmerman L., Wetzels S., Biessen E., Kersten S. (2022). Triglyceride breakdown from lipid droplets regulates the inflammatory response in macrophages. Proc. Natl. Acad. Sci. USA.

[42] Koshy A.N., Nerlekar N., Gow P.J., Lim R., Smith G., Galea M., Rodrigues T.S., Lim H.S., Teh A.W., Farouque O. (2022). A prospective natural history study of coronary atherosclerosis following liver transplantation. Atherosclerosis.

[43] Yang B., Hang S., Xu S., Gao Y., Yu W., Zang G., Zhang L., Wang Z. (2024). Macrophage polarisation and inflammatory mechanisms in atherosclerosis: Implications for prevention and treatment. Heliyon.

[44] Yurdagul A., Subramanian M., Wang X., Crown S.B., Ilkayeva O.R., Darville L., Kolluru G.K., Rymond C.C., Gerlach B.D., Zheng Z. (2020). Macrophage Metabolism of Apoptotic Cell-Derived Arginine Promotes Continual Efferocytosis and Resolution of Injury. Cell Metab..

[45] Jinnouchi H., Guo L., Sakamoto A., Torii S., Sato Y., Cornelissen A., Kuntz S., Paek K.H., Fernandez R., Fuller D. (2020). Diversity of macrophage phenotypes and responses in atherosclerosis. Cell Mol. Life Sci..

[46] Rahman K., Vengrenyuk Y., Ramsey S.A., Vila N.R., Girgis N.M., Liu J., Gusarova V., Gromada J., Weinstock A., Moore K.J. (2017). Inflammatory Ly6Chi monocytes and their conversion to M2 macrophages drive atherosclerosis regression. J. Clin. Investig..

[47] Auffray C., Sieweke M.H., Geissmann F. (2009). Blood monocytes: Development, heterogeneity, and relationship with dendritic cells. Annu. Rev. Immunol..

[48] Van Dyken S.J., Locksley R.M. (2013). Interleukin-4- and interleukin-13-mediated alternatively activated macrophages: Roles in homeostasis and disease. Annu. Rev. Immunol..

[49] Cheng P., Wirka R.C., Shoa Clarke L., Zhao Q., Kundu R., Nguyen T., Nair S., Sharma D., Kim H.J., Shi H. (2022). ZEB2 Shapes the Epigenetic Landscape of Atherosclerosis. Circulation.

[50] Winkels H., Ehinger E., Vassallo M., Buscher K., Dinh H.Q., Kobiyama K., Hamers A.A.J., Cochain C., Vafadarnejad E., Saliba A.E. (2018). Atlas of the Immune Cell Repertoire in Mouse Atherosclerosis Defined by Single-Cell RNA-Sequencing and Mass Cytometry. Circ. Res..

[51] Tan J., Liang Y., Yang Z., He Q., Tong J., Deng Y., Guo W., Liang K., Tang J., Shi W. (2023). Single-Cell Transcriptomics Reveals Crucial Cell Subsets and Functional Heterogeneity Associated With Carotid Atherosclerosis and Cerebrovascular Events. Arterioscler. Thromb. Vasc. Biol..

[52] Meng X., Yang J., Dong M., Zhang K., Tu E., Gao Q., Chen W., Zhang C., Zhang Y. (2016). Regulatory T cells in cardiovascular diseases. Nat. Rev. Cardiol..

[53] Ou H.X., Guo B.B., Liu Q., Li Y.K., Yang Z., Feng W.J., Mo Z.C. (2018). Regulatory T cells as a new therapeutic target for atherosclerosis. Acta Pharmacol. Sin..

[54] Hansson G.K., Hermansson A. (2011). The immune system in atherosclerosis. Nat. Immunol..

[55] Weng X., Cheng X., Wu X., Xu H., Fang M., Xu Y. (2014). Sin3B mediates collagen type I gene repression by interferon gamma in vascular smooth muscle cells. Biochem. Biophys. Res. Commun..

[56] Li N., McLaren J.E., Michael D.R., Clement M., Fielding C.A., Ramji D.P. (2010). ERK is integral to the IFN-gamma-mediated activation of STAT1, the expression of key genes implicated in atherosclerosis, and the uptake of modified lipoproteins by human macrophages. J. Immunol..

[57] Brauner S., Jiang X., Thorlacius G.E., Lundberg A.M., Ostberg T., Yan Z.Q., Kuchroo V.K., Hansson G.K., Wahren-Herlenius M. (2018). Augmented Th17 differentiation in Trim21 deficiency promotes a stable phenotype of atherosclerotic plaques with high collagen content. Cardiovasc. Res..

[58] Akhavanpoor M., Akhavanpoor H., Gleissner C.A., Wangler S., Doesch A.O., Katus H.A., Erbel C. (2017). The Two Faces of Interleukin-17A in Atherosclerosis. Curr. Drug Targets.

[59] De Palma R., Del Galdo F., Abbate G., Chiariello M., Calabro R., Forte L., Cimmino G., Papa M.F., Russo M.G., Ambrosio G. (2006). Patients with acute coronary syndrome show oligoclonal T-cell recruitment within unstable plaque: Evidence for a local, intracoronary immunologic mechanism. Circulation.

[60] Pi H., Wang G., Wang Y., Zhang M., He Q., Zheng X., Yin K., Zhao G., Jiang T. (2024). Immunological perspectives on atherosclerotic plaque formation and progression. Front. Immunol..

[61] Kyaw T., Tay C., Krishnamurthi S., Kanellakis P., Agrotis A., Tipping P., Bobik A., Toh B.H. (2011). B1a B lymphocytes are atheroprotective by secreting natural IgM that increases IgM deposits and reduces necrotic cores in atherosclerotic lesions. Circ. Res..

[62] Caligiuri G., Nicoletti A., Poirier B., Hansson G.K. (2002). Protective immunity against atherosclerosis carried by B cells of hypercholesterolemic mice. J. Clin. Investig..

[63] Upadhye A., Srikakulapu P., Gonen A., Hendrikx S., Perry H.M., Nguyen A., McSkimming C., Marshall M.A., Garmey J.C., Taylor A.M. (2019). Diversification and CXCR4-Dependent Establishment of the Bone Marrow B-1a Cell Pool Governs Atheroprotective IgM Production Linked to Human Coronary Atherosclerosis. Circ. Res..

[64] Pan Z., Zhu T., Liu Y., Zhang N. (2022). Role of the CXCL13/CXCR5 Axis in Autoimmune Diseases. Front. Immunol..

[65] Yin C., Mohanta S.K., Srikakulapu P., Weber C., Habenicht A.J. (2016). Artery Tertiary Lymphoid Organs: Powerhouses of Atherosclerosis Immunity. Front. Immunol..

[66] Hill C.E., Rummery N., Hickey H., Sandow S.L. (2002). Heterogeneity in the distribution of vascular gap junctions and connexins: Implications for function. Clin. Exp. Pharmacol. Physiol..

[67] Volz K.S., Jacobs A.H., Chen H.I., Poduri A., McKay A.S., Riordan D.P., Kofler N., Kitajewski J., Weissman I., Red-Horse K. (2015). Pericytes are progenitors for coronary artery smooth muscle. eLife.

[68] Sabbineni H., Verma A., Somanath P.R. (2018). Isoform-specific effects of transforming growth factor beta on endothelial-to-mesenchymal transition. J. Cell Physiol..

[69] Battig M.R., Fishbein I., Levy R.J., Alferiev I.S., Guerrero D., Chorny M. (2018). Optimizing endothelial cell functionalization for cell therapy of vascular proliferative disease using a direct contact co-culture system. Drug Deliv. Transl. Res..

[70] Ying R., Li S.W., Chen J.Y., Zhang H.F., Yang Y., Gu Z.J., Chen Y.X., Wang J.F. (2018). Endoplasmic reticulum stress in perivascular adipose tissue promotes destabilization of atherosclerotic plaque by regulating GM-CSF paracrine. J. Transl. Med..

[71] Zhang Z., Yi D., Zhou J., Zheng Y., Gao Z., Hu X., Ying G., Peng X., Wen T. (2020). Exosomal LINC01005 derived from oxidized low-density lipoprotein-treated endothelial cells regulates vascular smooth muscle cell phenotypic switch. Biofactors.

[72] Song T., Zhao S., Luo S., Chen C., Liu X., Wu X., Sun Z., Cao J., Wang Z., Wang Y. (2024). SLC44A2 regulates vascular smooth muscle cell phenotypic switching and aortic aneurysm. J. Clin. Investig..

[73] Jia Y., Mao C., Ma Z., Huang J., Li W., Ma X., Zhang S., Li M., Yu F., Sun Y. (2022). PHB2 Maintains the Contractile Phenotype of VSMCs by Counteracting PKM2 Splicing. Circ. Res..

[74] Ben Dhaou C., Al-Yafeai Z., Cruz-Marquez G.A., Scott M.L., Pearson-Gallion B., Cockerham E., Li H., Peretik J.M., Hong Y., Dhanesha N. (2025). Integrin-specific signaling drives ER stress-dependent atherogenic endothelial activation. Redox Biol..

[75] Shimizu T., Maruyama K., Kawamura T., Urade Y., Wada Y. (2020). PERK participates in cardiac valve development via fatty acid oxidation and endocardial-mesenchymal transformation. Sci. Rep..

[76] Chung J., Kim K.H., Lee S.C., An S.H., Kwon K. (2015). Ursodeoxycholic Acid (UDCA) Exerts Anti-Atherogenic Effects by Inhibiting Endoplasmic Reticulum (ER) Stress Induced by Disturbed Flow. Mol. Cells.

[77] Larsen J.H., Hegelund J.S., Pedersen M.K., Andersson C.M., Lindegaard C.A., Hansen D.R., Stubbe J., Lindholt J.S., Hansen C.S., Grentzmann A. (2024). Smooth muscle-specific deletion of cellular communication network factor 2 causes severe aorta malformation and atherosclerosis. Cardiovasc. Res..

[78] Aranguiz P., Romero P., Vasquez F., Flores-Vergara R., Aravena D., Sanchez G., Gonzalez M., Olmedo I., Pedrozo Z. (2021). Polycystin-1 mitigates damage and regulates CTGF expression through AKT activation during cardiac ischemia/reperfusion. Biochim. Biophys. Acta Mol. Basis Dis..

[79] Li S., Cao S., Li P., Zhang F., Ren G., Wang X., Zhao J., Liu C., Gao Y., Xu J. (2026). Endothelial BMP6 Drives Hemodynamic-Dependent VSMCs Calcification in Carotid Atherosclerosis. Adv. Sci..

[80] Jia L., Wang L., Wei F., Li C., Wang Z., Yu H., Chen H., Wang B., Jiang A. (2020). Effects of Caveolin-1-ERK1/2 pathway on endothelial cells and smooth muscle cells under shear stress. Exp. Biol. Med..

[81] Yalim Z., Onrat S.T., Dural I.E., Onrat E. (2023). Could Aneurysm and Atherosclerosis-Associated MicroRNAs (miR 24-1-5p, miR 34a-5p, miR 126-5p, miR 143-5p, miR 145-5p) Also Be Associated with Coronary Artery Ectasia?. Genet. Test. Mol. Biomark..

[82] Zhang M., Li F., Wang X., Gong J., Xian Y., Wang G., Zheng Z., Shang C., Wang B., He Y. (2020). MiR-145 alleviates Hcy-induced VSMC proliferation, migration, and phenotypic switch through repression of the PI3K/Akt/mTOR pathway. Histochem. Cell Biol..

[83] Fish J.E., Santoro M.M., Morton S.U., Yu S., Yeh R.F., Wythe J.D., Ivey K.N., Bruneau B.G., Stainier D.Y., Srivastava D. (2008). miR-126 regulates angiogenic signaling and vascular integrity. Dev. Cell.

[84] Song Y., Wang T., Mu C., Gui W., Deng Y., Ma R. (2022). LncRNA SENCR overexpression attenuated the proliferation, migration and phenotypic switching of vascular smooth muscle cells in aortic dissection via the miR-206/myocardin axis. Nutr. Metab. Cardiovasc. Dis..

[85] Huang C., Fang X., Xie X., Liu Y., Xu D., Meng X., Long J. (2021). Effect of miR-126 on the Proliferation and Migration of Vascular Smooth Muscle Cells in Aortic Aneurysm Mice Under PI3K/AKT/mTOR Signaling Pathway. Mol. Biotechnol..

[86] Song T., Zhou M., Li W., Zheng L., Wu J., Zhao M. (2022). Tripeptide Leu-Ser-Trp Regulates the Vascular Endothelial Cells Phenotype Switching by Mediating the Vascular Smooth Muscle Cells-Derived Small Extracellular Vesicles Packaging of miR-145. Molecules.

[87] Patel N., Chin D.D., Magee G.A., Chung E.J. (2022). Therapeutic Response of miR-145 Micelles on Patient-Derived Vascular Smooth Muscle Cells. Front. Digit. Health.

[88] Hergenreider E., Heydt S., Treguer K., Boettger T., Horrevoets A.J., Zeiher A.M., Scheffer M.P., Frangakis A.S., Yin X., Mayr M. (2012). Atheroprotective communication between endothelial cells and smooth muscle cells through miRNAs. Nat. Cell Biol..

[89] Zhou Z., Schober A., Nazari-Jahantigh M. (2018). Dicer promotes endothelial recovery and limits lesion formation after vascular injury through miR-126-5p. Int. J. Cardiol..

[90] Chen T., Sun M.R., Zhou Q., Guzman A.M., Ramchandran R., Chen J., Fraidenburg D.R., Ganesh B., Maienschein-Cline M., Obrietan K. (2022). MicroRNA-212-5p, an anti-proliferative miRNA, attenuates hypoxia and sugen/hypoxia-induced pulmonary hypertension in rodents. Mol. Ther. Nucleic Acids.

[91] Li L., Mao D., Li C., Li M. (2018). miR-145-5p Inhibits Vascular Smooth Muscle Cells (VSMCs) Proliferation and Migration by Dysregulating the Transforming Growth Factor-b Signaling Cascade. Med. Sci. Monit..

[92] Alaqeeli M., Mayaki D., Hussain S.N.A. (2021). Long Non-coding RNA Rhabdomyosarcoma 2-Associated Transcript Regulates Angiogenesis in Endothelial Cells. Front. Physiol..

[93] Huang J., Lin S.C., Nadershahi A., Watts S.W., Sarkar R. (2008). Role of redox signaling and poly (adenosine diphosphate-ribose) polymerase activation in vascular smooth muscle cell growth inhibition by nitric oxide and peroxynitrite. J. Vasc. Surg..

[94] Li L., Wang Z., Hu X., Wan T., Wu H., Jiang W., Hu R. (2016). Human aortic smooth muscle cell-derived exosomal miR-221/222 inhibits autophagy via a PTEN/Akt signaling pathway in human umbilical vein endothelial cells. Biochem. Biophys. Res. Commun..

[95] Toton-Zuranska J., Mikolajczyk T.P., Saju B., Guzik T.J. (2024). Vascular remodelling in cardiovascular diseases: Hypertension, oxidation, and inflammation. Clin. Sci..

[96] Hamczyk M.R., Nevado R.M., Gonzalo P., Andres-Manzano M.J., Nogales P., Quesada V., Rosado A., Torroja C., Sanchez-Cabo F., Dopazo A. (2024). Endothelial-to-Mesenchymal Transition Contributes to Accelerated Atherosclerosis in Hutchinson-Gilford Progeria Syndrome. Circulation.

[97] Lo H.M., Hwang T.L., Wu W.B. (2017). A Phenanthrene Derivative, 5,7-Dimethoxy-1,4-Phenanthrenequinone, Inhibits Cell Adhesion Molecule Expression and Migration in Vascular Endothelial and Smooth Muscle Cells. Pharmacology.

[98] Alrahimi J.S., Ahmed F.A., Haneef A.A., Atar D. (2025). Integrative Perspectives on Atherosclerosis: From Molecular Mechanisms to Therapeutic Approaches. Saudi J. Med. Med. Sci..

[99] Jebari-Benslaiman S., Galicia-Garcia U., Larrea-Sebal A., Olaetxea J.R., Alloza I., Vandenbroeck K., Benito-Vicente A., Martin C. (2022). Pathophysiology of Atherosclerosis. Int. J. Mol. Sci..

[100] Bonacina F., Da Dalt L., Catapano A.L., Norata G.D. (2021). Metabolic adaptations of cells at the vascular-immune interface during atherosclerosis. Mol. Asp. Med..

[101] Inoue M., Itoh H., Tanaka T., Chun T.H., Doi K., Fukunaga Y., Sawada N., Yamshita J., Masatsugu K., Saito T. (2001). Oxidized LDL regulates vascular endothelial growth factor expression in human macrophages and endothelial cells through activation of peroxisome proliferator-activated receptor-gamma. Arterioscler. Thromb. Vasc. Biol..

[102] Chang Y.J., Li Y.S., Wu C.C., Wang K.C., Huang T.C., Chen Z., Chien S. (2019). Extracellular MicroRNA-92a Mediates Endothelial Cell-Macrophage Communication. Arterioscler. Thromb. Vasc. Biol..

[103] He S., Wu C., Xiao J., Li D., Sun Z., Li M. (2018). Endothelial extracellular vesicles modulate the macrophage phenotype: Potential implications in atherosclerosis. Scand. J. Immunol..

[104] Shao D., Lian Z., Di Y., Zhang L., Rajoka M.S.R., Zhang Y., Kong J., Jiang C., Shi J. (2018). Dietary compounds have potential in controlling atherosclerosis by modulating macrophage cholesterol metabolism and inflammation via miRNA. npj Sci. Food.

[105] Oberoi R., Schuett J., Schuett H., Koch A.K., Luchtefeld M., Grote K., Schieffer B. (2016). Targeting Tumor Necrosis Factor-alpha with Adalimumab: Effects on Endothelial Activation and Monocyte Adhesion. PLoS ONE.

[106] Karunakaran D., Nguyen M.A., Geoffrion M., Vreeken D., Lister Z., Cheng H.S., Otte N., Essebier P., Wyatt H., Kandiah J.W. (2021). RIPK1 Expression Associates with Inflammation in Early Atherosclerosis in Humans and Can Be Therapeutically Silenced to Reduce NF-kappaB Activation and Atherogenesis in Mice. Circulation.

[107] Zhang B., Yang Y., Yi J., Zhao Z., Ye R. (2021). Hyperglycemia modulates M1/M2 macrophage polarization via reactive oxygen species overproduction in ligature-induced periodontitis. J. Periodontal Res..

[108] Schulz C., Schafer A., Stolla M., Kerstan S., Lorenz M., von Bruhl M.L., Schiemann M., Bauersachs J., Gloe T., Busch D.H. (2007). Chemokine fractalkine mediates leukocyte recruitment to inflammatory endothelial cells in flowing whole blood: A critical role for P-selectin expressed on activated platelets. Circulation.

[109] Spehar Uroic A., Filipovic M., Sucur A., Kelava T., Kovacic N., Grcevic D. (2024). Distinct association patterns of chemokine profile and cardiometabolic status in children and adolescents with type 1 diabetes and obesity. Front. Endocrinol..

[110] Forteza M.J., Polyzos K.A., Baumgartner R., Suur B.E., Mussbacher M., Johansson D.K., Hermansson A., Hansson G.K., Ketelhuth D.F.J. (2018). Activation of the Regulatory T-Cell/Indoleamine 2,3-Dioxygenase Axis Reduces Vascular Inflammation and Atherosclerosis in Hyperlipidemic Mice. Front. Immunol..

[111] Chee Y.J., Dalan R., Cheung C. (2025). The Interplay Between Immunity, Inflammation and Endothelial Dysfunction. Int. J. Mol. Sci..

[112] Yu L., Yin M., Yang X., Lu M., Tang F., Wang H. (2018). Calpain inhibitor I attenuates atherosclerosis and inflammation in atherosclerotic rats through eNOS/NO/NF-kappaB pathway. Can. J. Physiol. Pharmacol..

[113] Jadli A.S., Parasor A., Gomes K.P., Shandilya R., Patel V.B. (2021). Exosomes in Cardiovascular Diseases: Pathological Potential of Nano-Messenger. Front. Cardiovasc. Med..

[114] De Caterina R., Bourcier T., Laufs U., La Fata V., Lazzerini G., Neish A.S., Libby P., Liao J.K. (2001). Induction of endothelial-leukocyte interaction by interferon-gamma requires coactivation of nuclear factor-kappaB. Arterioscler. Thromb. Vasc. Biol..

[115] Zhang H., Chen J., Liu X., Awar L., McMickle A., Bai F., Nagarajan S., Yu S. (2013). IL-17 induces expression of vascular cell adhesion molecule through signalling pathway of NF-kappaB, but not Akt1 and TAK1 in vascular smooth muscle cells. Scand. J. Immunol..

[116] Kim W.J., Kang Y.J., Suk K., Park J.E., Kwon B.S., Lee W.H. (2008). Comparative analysis of the expression patterns of various TNFSF/TNFRSF in atherosclerotic plaques. Immunol. Investig..

[117] Skoura A., Michaud J., Im D.S., Thangada S., Xiong Y., Smith J.D., Hla T. (2011). Sphingosine-1-phosphate receptor-2 function in myeloid cells regulates vascular inflammation and atherosclerosis. Arterioscler. Thromb. Vasc. Biol..

[118] Yaghooti H., Firoozrai M., Fallah S., Khorramizadeh M.R. (2010). Angiotensin II Differentially Induces Matrix Metalloproteinase-9 and Tissue Inhibitor of Metalloproteinase-1 Production and Disturbs MMP/TIMP Balance. Avicenna J. Med. Biotechnol..

[119] Lacy M., Burger C., Shami A., Ahmadsei M., Winkels H., Nitz K., van Tiel C.M., Seijkens T.T.P., Kusters P.J.H., Karshovka E. (2021). Cell-specific and divergent roles of the CD40L-CD40 axis in atherosclerotic vascular disease. Nat. Commun..

[120] Luo J.Y., Cheng C.K., He L., Pu Y., Zhang Y., Lin X., Xu A., Lau C.W., Tian X.Y., Ma R.C.W. (2022). Endothelial UCP2 Is a Mechanosensitive Suppressor of Atherosclerosis. Circ. Res..

[121] Khalyfa A., Zhang C., Khalyfa A.A., Foster G.E., Beaudin A.E., Andrade J., Hanly P.J., Poulin M.J., Gozal D. (2016). Effect on Intermittent Hypoxia on Plasma Exosomal Micro RNA Signature and Endothelial Function in Healthy Adults. Sleep.

[122] Qi F., Deng Y., Huang W., Cai Y., Hong K., Xiang S. (2024). Irisin suppresses PDGF-BB-induced proliferation of vascular smooth muscle cells in vitro by activating AMPK/mTOR-mediated autophagy. Eur. J. Histochem..

[123] Spivia W., Magno P.S., Le P., Fraser D.A. (2014). Complement protein C1q promotes macrophage anti-inflammatory M2-like polarization during the clearance of atherogenic lipoproteins. Inflamm. Res..

[124] Yan W., Li T., Yin T., Hou Z., Qu K., Wang N., Durkan C., Dong L., Qiu J., Gregersen H. (2020). M2 macrophage-derived exosomes promote the c-KIT phenotype of vascular smooth muscle cells during vascular tissue repair after intravascular stent implantation. Theranostics.

[125] Lu Q.B., Wan M.Y., Wang P.Y., Zhang C.X., Xu D.Y., Liao X., Sun H.J. (2018). Chicoric acid prevents PDGF-BB-induced VSMC dedifferentiation, proliferation and migration by suppressing ROS/NFkappaB/mTOR/P70S6K signaling cascade. Redox Biol..

[126] Robichaud S., Rasheed A., Pietrangelo A., Doyoung Kim A., Boucher D.M., Emerton C., Vijithakumar V., Gharibeh L., Fairman G., Mak E. (2022). Autophagy Is Differentially Regulated in Leukocyte and Nonleukocyte Foam Cells During Atherosclerosis. Circ. Res..

[127] Ostriker A., Horita H.N., Poczobutt J., Weiser-Evans M.C., Nemenoff R.A. (2014). Vascular smooth muscle cell-derived transforming growth factor-beta promotes maturation of activated, neointima lesion-like macrophages. Arterioscler. Thromb. Vasc. Biol..

[128] Owsiany K.M., Deaton R.A., Soohoo K.G., Tram Nguyen A., Owens G.K. (2022). Dichotomous Roles of Smooth Muscle Cell-Derived MCP1 (Monocyte Chemoattractant Protein 1) in Development of Atherosclerosis. Arterioscler. Thromb. Vasc. Biol..

[129] Yu H., Douglas H.F., Wathieu D., Braun R.A., Edomwande C., Lightell D.J., Pham T., Klingenberg N.C., Bishop S.P., Khismatullin D.B. (2023). Diabetes is accompanied by secretion of pro-atherosclerotic exosomes from vascular smooth muscle cells. Cardiovasc. Diabetol..

[130] Schafer S., Gogiraju R., Rosch M., Kerstan Y., Beck L., Garbisch J., Saliba A.E., Gistera A., Hermanns H.M., Boon L. (2024). CD8(+) T Cells Drive Plaque Smooth Muscle Cell Dedifferentiation in Experimental Atherosclerosis. Arterioscler. Thromb. Vasc. Biol..

[131] Shi Z., Rockey D.C. (2010). Interferon-gamma-mediated inhibition of serum response factor-dependent smooth muscle-specific gene expression. J. Biol. Chem..

[132] Cheng W.H., Wang Y. (2025). Inflammatory Pathways in Coronary Artery Disease: Which Ones to Target for Secondary Prevention?. Cells.

[133] Legare J.F., Issekutz T., Lee T.D., Hirsch G. (2000). CD8+ T lymphocytes mediate destruction of the vascular media in a model of chronic rejection. Am. J. Pathol..

[134] Sun P., Tang L.N., Li G.Z., Xu Z.L., Xu Q.H., Wang M., Li L. (2019). Effects of MiR-21 on the proliferation and migration of vascular smooth muscle cells in rats with atherosclerosis via the Akt/ERK signaling pathway. Eur. Rev. Med. Pharmacol. Sci..

[135] Stralin P., Marklund S.L. (2000). Multiple cytokines regulate the expression of extracellular superoxide dismutase in human vascular smooth muscle cells. Atherosclerosis.

[136] Furmanik M., Chatrou M., van Gorp R., Akbulut A., Willems B., Schmidt H., van Eys G., Bochaton-Piallat M.L., Proudfoot D., Biessen E. (2020). Reactive Oxygen-Forming Nox5 Links Vascular Smooth Muscle Cell Phenotypic Switching and Extracellular Vesicle-Mediated Vascular Calcification. Circ. Res..

[137] Wang K., Li W., Yu Q., Guo B., Yang B., Zhang C., Li M., Li J., Hu S., Zheng Q. (2017). High Mobility Group Box 1 Mediates Interferon-gamma-Induced Phenotypic Modulation of Vascular Smooth Muscle Cells. J. Cell Biochem..

[138] Gao Z., Xu X., Li Y., Sun K., Yang M., Zhang Q., Wang S., Lin Y., Lou L., Wu A. (2021). Mechanistic Insight into PPARgamma and Tregs in Atherosclerotic Immune Inflammation. Front. Pharmacol..

[139] Yang X., Coriolan D., Murthy V., Schultz K., Golenbock D.T., Beasley D. (2005). Proinflammatory phenotype of vascular smooth muscle cells: Role of efficient Toll-like receptor 4 signaling. Am. J. Physiol. Heart Circ. Physiol..

[140] Meletta R., Muller Herde A., Dennler P., Fischer E., Schibli R., Kramer S.D. (2016). Preclinical imaging of the co-stimulatory molecules CD80 and CD86 with indium-111-labeled belatacept in atherosclerosis. EJNMMI Res..

[141] Qiu X., Shi Q., Huang Y., Jiang H., Qin S. (2021). miR-143/145 inhibits Th9 cell differentiation by targeting NFATc1. Mol. Immunol..

[142] Gluchowska A., Cysewski D., Baj-Krzyworzeka M., Szatanek R., Weglarczyk K., Podszywalow-Bartnicka P., Sunderland P., Kozlowska E., Sliwinska M.A., Dabrowski M. (2022). Unbiased proteomic analysis of extracellular vesicles secreted by senescent human vascular smooth muscle cells reveals their ability to modulate immune cell functions. Geroscience.

[143] Buxade M., Huerga Encabo H., Riera-Borrull M., Quintana-Gallardo L., Lopez-Cotarelo P., Tellechea M., Martinez-Martinez S., Redondo J.M., Martin-Caballero J., Flores J.M. (2018). Macrophage-specific MHCII expression is regulated by a remote Ciita enhancer controlled by NFAT5. J. Exp. Med..

[144] Wigren M., Rattik S., Yao Mattisson I., Tomas L., Gronberg C., Soderberg I., Alm R., Sundius L., Ljungcrantz I., Bjorkbacka H. (2019). Lack of Ability to Present Antigens on Major Histocompatibility Complex Class II Molecules Aggravates Atherosclerosis in ApoE(-/-) Mice. Circulation.

[145] Lutgens E., Lievens D., Beckers L., Wijnands E., Soehnlein O., Zernecke A., Seijkens T., Engel D., Cleutjens J., Keller A.M. (2010). Deficient CD40-TRAF6 signaling in leukocytes prevents atherosclerosis by skewing the immune response toward an antiinflammatory profile. J. Exp. Med..

[146] Teng Y., Tao X., Lu W., Huang Y., Xu D., Li M., Fan Y. (2020). Identification of Hidradenitis Suppurativa-Related mRNA Expression Patterns Through Analysis of Gene Expression Omnibus. Dose Response.

[147] Reardon C.A., Lingaraju A., Schoenfelt K.Q., Zhou G., Cui C., Jacobs-El H., Babenko I., Hoofnagle A., Czyz D., Shuman H. (2018). Obesity and Insulin Resistance Promote Atherosclerosis through an IFNgamma-Regulated Macrophage Protein Network. Cell Rep..

[148] Ait-Oufella H., Salomon B.L., Potteaux S., Robertson A.K., Gourdy P., Zoll J., Merval R., Esposito B., Cohen J.L., Fisson S. (2006). Natural regulatory T cells control the development of atherosclerosis in mice. Nat. Med..

[149] Kim H.S., Woo J.S., Kim B.Y., Jang H.H., Hwang S.J., Kwon S.J., Choi E.Y., Kim J.B., Cheng X., Jin E. (2014). Biochemical and clinical correlation of intraplaque neovascularization using contrast-enhanced ultrasound of the carotid artery. Atherosclerosis.

[150] Oviedo-Orta E., Bermudez-Fajardo A., Karanam S., Benbow U., Newby A.C. (2008). Comparison of MMP-2 and MMP-9 secretion from T helper 0, 1 and 2 lymphocytes alone and in coculture with macrophages. Immunology.

[151] Lukowski S.W., Patel J., Andersen S.B., Sim S.L., Wong H.Y., Tay J., Winkler I., Powell J.E., Khosrotehrani K. (2019). Single-Cell Transcriptional Profiling of Aortic Endothelium Identifies a Hierarchy from Endovascular Progenitors to Differentiated Cells. Cell Rep..

[152] Depuydt M.A.C., Prange K.H.M., Slenders L., Ord T., Elbersen D., Boltjes A., de Jager S.C.A., Asselbergs F.W., de Borst G.J., Aavik E. (2020). Microanatomy of the Human Atherosclerotic Plaque by Single-Cell Transcriptomics. Circ. Res..

[153] Adelus M.L., Ding J., Tran B.T., Conklin A.C., Golebiewski A.K., Stolze L.K., Whalen M.B., Cusanovich D.A., Romanoski C.E. (2024). Single-cell ‘omic profiles of human aortic endothelial cells in vitro and human atherosclerotic lesions ex vivo reveal heterogeneity of endothelial subtype and response to activating perturbations. eLife.

[154] Wirka R.C., Wagh D., Paik D.T., Pjanic M., Nguyen T., Miller C.L., Kundu R., Nagao M., Coller J., Koyano T.K. (2019). Atheroprotective roles of smooth muscle cell phenotypic modulation and the TCF21 disease gene as revealed by single-cell analysis. Nat. Med..

[155] Bleckwehl T., Babler A., Tebens M., Maryam S., Nyberg M., Bosteen M., Halder M., Shaw I., Fleig S., Pyke C. (2025). Encompassing view of spatial and single-cell RNA sequencing renews the role of the microvasculature in human atherosclerosis. Nat. Cardiovasc. Res..

[156] Deng H., Sun Y., Zeng W., Li H., Guo M., Yang L., Lu B., Yu B., Fan G., Gao Q. (2020). New Classification of Macrophages in Plaques: A Revolution. Curr. Atheroscler. Rep..

[157] Willemsen L., de Winther M.P. (2020). Macrophage subsets in atherosclerosis as defined by single-cell technologies. J. Pathol..

[158] Patterson M.T., Firulyova M.M., Xu Y., Hillman H., Bishop C., Zhu A., Hickok G.H., Schrank P.R., Ronayne C.E., Caillot Z. (2023). Trem2 promotes foamy macrophage lipid uptake and survival in atherosclerosis. Nat. Cardiovasc. Res..

[159] Lu Y., Wu S., Zhu S., Shen J., Liu C., Zhao C., Su S., Ma H., Xiang M., Xie Y. (2024). Integrated Single-Cell Analysis Revealed Novel Subpopulations of Foamy Macrophages in Human Atherosclerotic Plaques. Biomolecules.

[160] Burger F., Baptista D., Roth A., Brandt K.J., da Silva R.F., Montecucco F., Mach F., Miteva K. (2022). Single-Cell RNA-Seq Reveals a Crosstalk between Hyaluronan Receptor LYVE-1-Expressing Macrophages and Vascular Smooth Muscle Cells. Cells.

[161] Koupenova M., Clancy L., Corkrey H.A., Freedman J.E. (2018). Circulating Platelets as Mediators of Immunity, Inflammation, and Thrombosis. Circ. Res..

[162] Warnatsch A., Ioannou M., Wang Q., Papayannopoulos V. (2015). Inflammation. Neutrophil extracellular traps license macrophages for cytokine production in atherosclerosis. Science.

[163] Quillard T., Araujo H.A., Franck G., Shvartz E., Sukhova G., Libby P. (2015). TLR2 and neutrophils potentiate endothelial stress, apoptosis and detachment: Implications for superficial erosion. Eur. Heart J..

[164] Pordzik J., Pisarz K., De Rosa S., Jones A.D., Eyileten C., Indolfi C., Malek L., Postula M. (2018). The Potential Role of Platelet-Related microRNAs in the Development of Cardiovascular Events in High-Risk Populations, Including Diabetic Patients: A Review. Front. Endocrinol..

[165] Pircher J., Engelmann B., Massberg S., Schulz C. (2019). Platelet-Neutrophil Crosstalk in Atherothrombosis. Thromb. Haemost..

[166] Tang J., Li T., Xiong X., Yang Q., Su Z., Zheng M., Chen Q. (2023). Colchicine delivered by a novel nanoparticle platform alleviates atherosclerosis by targeted inhibition of NF-kappaB/NLRP3 pathways in inflammatory endothelial cells. J. Nanobiotechnol..

[167] Zhou H., Khan D., Hussain S.M., Gerdes N., Hagenbeck C., Rana M., Cornelius J.F., Muhammad S. (2023). Colchicine prevents oxidative stress-induced endothelial cell senescence via blocking NF-kappaB and MAPKs: Implications in vascular diseases. J. Inflamm..

[168] Ikeda U., Shimpo M., Ohki R., Inaba H., Takahashi M., Yamamoto K., Shimada K. (2000). Fluvastatin inhibits matrix metalloproteinase-1 expression in human vascular endothelial cells. Hypertension.

[169] Sanyour H.J., Li N., Rickel A.P., Torres H.M., Anderson R.H., Miles M.R., Childs J.D., Francis K.R., Tao J., Hong Z. (2020). Statin-mediated cholesterol depletion exerts coordinated effects on the alterations in rat vascular smooth muscle cell biomechanics and migration. J. Physiol..

[170] Rodrigues-Diez R.R., Garcia-Redondo A.B., Orejudo M., Rodrigues-Diez R., Briones A.M., Bosch-Panadero E., Kery G., Pato J., Ortiz A., Salaices M. (2015). The C-terminal module IV of connective tissue growth factor, through EGFR/Nox1 signaling, activates the NF-kappaB pathway and proinflammatory factors in vascular smooth muscle cells. Antioxid. Redox Signal.

[171] Zhang Q., Ma S., Kang X., Liu Y., Ma F., Yu F., Luo X., Li G., Hao Y., Zhang H. (2025). A dual-targeting bio-liposomes nanodrug repair endothelial cell dysfunction and restore macrophage cholesterol flow homeostasis to treat early atherosclerosis. J. Nanobiotechnol..

[172] Safaeian L., Vaseghi G., Jabari H., Dana N. (2019). Evolocumab, a proprotein convertase subtilisin/kexin type 9 inhibitor, promotes angiogenesis in vitro. Can. J. Physiol. Pharmacol..

[173] Shin D., Kim S., Lee H., Lee H.C., Lee J., Park H.W., Fukai M., Choi E., Choi S., Koo B.J. (2024). PCSK9 stimulates Syk, PKCdelta, and NF-kappaB, leading to atherosclerosis progression independently of LDL receptor. Nat. Commun..

[174] Cimato T.R., Palka B.A. (2014). Fractalkine (CX3CL1), GM-CSF and VEGF-a levels are reduced by statins in adult patients. Clin. Transl. Med..

[175] Tan H., Liu L., Qi Y., Zhang D., Zhi Y., Li Y., Zhang H., Liu J. (2024). Atorvastatin Attenuates Endothelial Cell Injury in Atherosclerosis Through Inhibiting ACSL4-Mediated Ferroptosis. Cardiovasc. Ther..

[176] Nie P., Li D., Hu L., Jin S., Yu Y., Cai Z., Shao Q., Shen J., Yi J., Xiao H. (2014). Atorvastatin improves plaque stability in ApoE-knockout mice by regulating chemokines and chemokine receptors. PLoS ONE.

[177] Ba W., Xu Y., Yin G., Yang J., Wang R., Chi S., Wang Y., Li C. (2019). Metformin inhibits pro-inflammatory responses via targeting nuclear factor-kappaB in HaCaT cells. Cell Biochem. Funct..

[178] Huangfu N., Wang Y., Cheng J., Xu Z., Wang S. (2018). Metformin protects against oxidized low density lipoprotein-induced macrophage apoptosis and inhibits lipid uptake. Exp. Ther. Med..

[179] Ma Y., Zhang Y., Qiu C., He C., He T., Shi S., Liu Z. (2021). Rivaroxaban Suppresses Atherosclerosis by Inhibiting FXa-Induced Macrophage M1 Polarization-Mediated Phenotypic Conversion of Vascular Smooth Muscle Cells. Front. Cardiovasc. Med..

[180] Lutgens E., Cleutjens K.B., Heeneman S., Koteliansky V.E., Burkly L.C., Daemen M.J. (2000). Both early and delayed anti-CD40L antibody treatment induces a stable plaque phenotype. Proc. Natl. Acad. Sci. USA.

[181] Zhong W., Kokhaei P., Mulder T.A., Ghaderi A., Moshfegh A., Lundin J., Palma M., Schultz J., Olin T., Osterborg A. (2024). A Small Molecule Antagonist of CX3CR1 (KAND567) Inhibited the Tumor Growth-Promoting Effect of Monocytes in Chronic Lymphocytic Leukemia (CLL). Cancers.

[182] Dong L., Nordlohne J., Ge S., Hertel B., Melk A., Rong S., Haller H., von Vietinghoff S. (2016). T Cell CX3CR1 Mediates Excess Atherosclerotic Inflammation in Renal Impairment. J. Am. Soc. Nephrol..

[183] Hwang A.R., Nam J.O., Kang Y.J. (2018). Fluvastatin inhibits advanced glycation end products-induced proliferation, migration, and extracellular matrix accumulation in vascular smooth muscle cells by targeting connective tissue growth factor. Korean J. Physiol. Pharmacol..

[184] Zhu L.Y., Xue C., Bashore A.C., Cui J., Yao S., Sachs N., Wettich J.L., Coronel J., Garcia E.J., Khalid S. (2026). PERK Is Dispensable for Smooth Muscle Cell Phenotype Switching in Atherosclerosis. Arterioscler. Thromb. Vasc. Biol..

[185] Mazan-Mamczarz K., Tsitsipatis D., Childs B.G., Carr A.E., Dos Santos C.R., Anerillas C., Romero B., Gregg J.M., Henry-Smith C., Okereke A.N. (2025). Single-cell and spatial transcriptomics map senescent vascular cells in arterial remodeling during atherosclerosis in mice. Nat. Aging.

[186] Sun J., Singh P., Shami A., Kluza E., Pan M., Djordjevic D., Michaelsen N.B., Kennback C., van der Wel N.N., Orho-Melander M. (2023). Spatial Transcriptional Mapping Reveals Site-Specific Pathways Underlying Human Atherosclerotic Plaque Rupture. J. Am. Coll. Cardiol..

[187] Pauli J., Garger D., Peymani F., Wettich J., Sachs N., Wirth J., Steiger K., Hillig C., Zhang H., Tabas I. (2025). Single cell spatial transcriptomics integration deciphers the morphological heterogeneity of atherosclerotic carotid arteries. Nat. Commun..

[188] Wang Y., Liu X.Y., Wang Y., Zhao W.X., Li F.D., Guo P.R., Fan Q., Wu X.F. (2023). NOX2 inhibition stabilizes vulnerable plaques by enhancing macrophage efferocytosis via MertK/PI3K/AKT pathway. Redox Biol..

